# Tetracycline Water Soluble Formulations with Enhanced Antimicrobial Activity

**DOI:** 10.3390/antibiotics9120845

**Published:** 2020-11-26

**Authors:** A. Meretoudi, C. N. Banti, P. Siafarika, A. G. Kalampounias, S. K. Hadjikakou

**Affiliations:** 1 Inorganic Chemistry Laboratory, Department of Chemistry, University of Ioannina, 45110 Ioannina, Greece; ameretoudi1996@gmail.com; 2 Physical Chemistry Laboratory, Department of Chemistry, University of Ioannina, 45110 Ioannina, Greece; pch1246@uoi.gr; 3 Institute of Materials Science and Computing, University Research Center of Ioannina (URCI), 45110 Ioannina, Greece

**Keywords:** biological inorganic chemistry, antibiotic, tetracycline, micelles, antimicrobial activity, toxicity

## Abstract

The negligible water solubility of tetracycline (**TC**), a well-known antibiotic of clinical use, is the major disadvantage for its oral administration. With the aim to improve the water solubility of **TC,** the micelles of formulae **SLS@TC** and **CTAB@TC** (**SLS** = sodium lauryl sulphate and **CTAB** = cetrimonium bromide) were synthesized. The micelles **SLS@TC** and **CTAB@TC** were characterized by melting point (m.p.), thermogravimetric differential thermal analysis (TG-DTA), differential scanning calorimetry (DTG/DSC), attenuated total reflection spectroscopy (FT-IR-ATR), ultra-violet visible (UV/vis) spectroscopy, proton nucleus magnetic resonance (^1^H-NMR) spectroscopy, and the ultrasonically-induced biregringence technique. The antimicrobial activity of **SLS@TC** and **CTAB@TC** was evaluated, by means of minimum inhibitory concentration (MIC), minimum bactericidal concentration (MBC), and inhibition zone (IZ), against the Gram negative bacterial strains *Pseudomonas aeruginosa* (*P. aeruginosa*) and *Escherichia coli (E. coli)* and the Gram positive ones of the genus *of Staphylococcus epidermidis* (S. *epidermidis*) and *Staphylococcus aureus* (*S. aureus*). Generally, both micelles show better activity than that of **TC** against the microbial strains tested. Thus, the MIC value of **CTAB@TC** is 550-fold higher than that of free **TC** against *S. epidermidis*. Despite the stronger activity of **CTAB@TC** than **SLS@TC** against both Gram negative and Gram positive microbes, **SLS@TC** is classified as a bactericidal agent (in that it eliminates 99.9% of the microbes), in contrast to **CTAB@TC**, which is bacteriostatic one (inhibits, but does not kill the organisms). The toxicity of **SLS@TC** and **CTAB@TC** was evaluated against human corneal eukaryotic cells (HCECs). Moreover, **SLS@TC** and **CTAB@TC** exhibit low in vivo toxicity against *Artemia salina*, even at concentrations up to threefold higher than those of their MIC^max^. Therefore, **SLS@TC** and **CTAB@TC** can be candidates for the development of new antibiotics.

## 1. Introduction

The properties of tetracycline (**TC**) are considered as ideal for antibiotic drugs [1]. **TC** is used for the treatment of a number of infections caused by both aerobic and anaerobic Gram positive and negative bacteria [2]. Its clinical safety and acceptable tolerability is proven [1]. Moreover, **TC** offers a valuable and relatively safe therapeutic modality in the treatment or inhibition of corneal ulceration [3]. However, the antimicrobial activity of **TC** suffers with resistance developed by microbes [1]. Moreover, the activity of **TC** is limited by the first passage phenomenon, where a portion of the antibiotic is always neutralized upon entering the gastrointestinal tract by stomach enzymes, bacteria, and enzymes of intestines and liver cells [4]. Thus, the development of new, non-toxic, water soluble formulations of **TC** for its administration through non gastrointestinal tract such as eye drops is a research, technological, and financial issue of great importance. One of the strategies used in order to overcome the low water solubility of **TC** is the formation of its hydrochloric salt [5]. However, this formulation affects the stomach acidity in the case of its oral administration, while it prevents its use as, e.g., eye drops. Solubilization of hydrophobic antibiotics by the use of surfactants is one of the strategies used to enhance their bioavailability [6,7,8,9,10,11,12,13,14,15].

Bacterial conjunctivitis, on the other hand, is an eye disease, which is related to inflammation of the outer lining of the eye and the inner surface of the eyelid [16]. Bacterial conjunctivitis is caused by microorganisms such as *Staphylococcus aureus*, *Haemophilus influenzae*, *Streptococcus pneumoniae*, and *Pseudomonas aeruginosa* [17]. Nowadays, the treatment of bacterial conjunctivitis is based on penicillin and rarely on aminoglycosides (gentamicine, trompainkine, and neomycine) and fluoroquinolones (ofloxacin and norfloxacine) [17]. However, therapy with these antibiotics suffers from many disadvantages, e.g., ineffectiveness of peniciline against Gram negative microbes [18], high toxicity of aminoglycoside, [19], and resistance to fluoroquinolones [20].

**Scheme 1:**
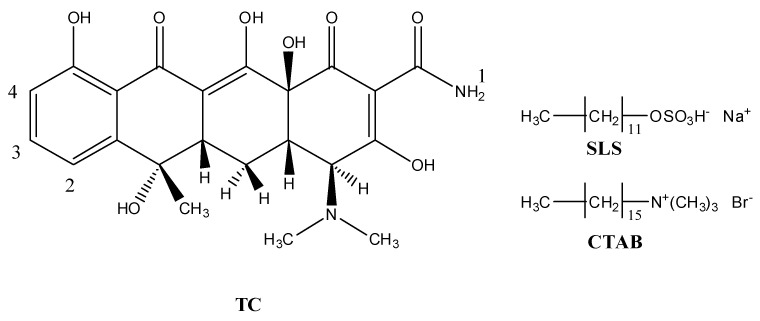
Molecular diagrams of tetracycline (**TC**), sodium lauryl sulfate (**SLS**), and cetrimonium bromide (**CTAB**).

In the course of our studies towards the development of metallotherapeutic [21,22,23,24,25,26,27,28,29], the water soluble micelles **SLS@TC** and **CTAB@TC** (**TC** = tetracycline, **SLS** = sodium lauryl sulfate, **CTAB** = cetrimonium bromide (Scheme 1)) were synthesized and characterized by thermogravimetric differential thermal analysis (TG-DTA), differential scanning calorimetry (DTG/DSC), and attenuated total reflection spectroscopy (FT-IR-ATR), UV/vis, proton nucleus magnetic resonance (^1^H-NMR), and the ultrasonically-induced birefringence technique. The minimum inhibitory concentration (MIC), minimum bactericidal concentration (MBC), and inhibition zone (IZ) of the micelles against *P. aeruginosa*, *E. coli*, *S. epidermidis*, and *S. aureus* were evaluated. The in vitro toxicity of the new formulations was determined against human corneal eukaryotic cells (HCECs). The in vivo toxicity was tested against *Artemia salina*. The **SLS** and **CTAB** were chosen for the solubilazation of **TC** because (i) both are used for drug delivery [30,31,32,33,34]; (ii) **CTAB** cation is an effective antiseptic agent against bacteria and fungi [35]; and (iii) SLS is an anionic body detergents-cleansers (shower gels and toothpastes) ingredient, without any harmful irritations [36], while United States Food and Drug Administration (FDA or USFDA) recognized it as a harmless food ingredient (21 CFR 172,822). Their usage is limited, however, by their irritating side effect, especially upon their administration to the eye. This disadvantage, on the other hand, is overcome by the high, nonirritant concentration (<0.1% *w/w*), which allows their use for the treatment or inhibition of corneal ulceration [36].

## 2. Results

### 2.1. Preparation and Characterization of Micelle

#### Determination of Critical Micelle Concentration (CMC)

Because of the low solubility of **TC** in aqueous media, the micelles **SLS@TC** and **CTAB@TC** are formed, using the **SLS** and **CTAB** as surfactants. In order to assert the formation of the **SLS@TC** and **CTAB@TC**, the determination of the CMC was performed via potentiometric titration at 36 °C [37] and the graph of conductivity values versus [**SLS**]/[**TC**] or [**CTAB**]/[**TC**] was drawn (Figure S1). Above the CMC value, surfactant molecules self-agglomerate to form the micelles [27]. Upon the formation of the micelles, a turning point (change of slope) is observed in the graph [27]. The CMC values were determined as follows: a solution of **SLS** or **CTAB** (2 × 10^−1^ M) in ddH_2_O was added to 20 mL ddH_2_O suspension of **TC** (1 × 10^−3^ M) in portions of 0.5 mL, while the conductivity values were subsequently recorded. The CMC value is obtained at 17/1 or 16/1, for [**SLS**]/[**TC**] or [**CTAB**]/[**TC**] molar ratios, respectively (Figure S1). These values lead to a content of 8.3% *w*/*w* **TC** in **SLS@TC** and 7.6% *w*/*w* **TC** in **CTAB@TC**. Both micelles **SLS@TC** and **CTAB@TC** are highly soluble in dd water and dimethyl sulfoxide (DMSO).

### 2.2. Solid State Studies

#### 2.2.1. Thermo Gravimetric Analysis of **SLS@TC** and **CTAB@TC**

##### Thermal Decomposition

TG/DTA analysis was performed under nitrogen. The micelle **SLS@TC** decomposes with three exothermic steps at 157.0–227.2 °C (mass loss: −0.92%) because of the elimination of water, at 227.2–292.1 °C (mass loss: −62.95%) and 292.1–500 °C (mass loss: −3.75%) °C (Figure S2A), with a total mass loss of 67.67%. The **SLS** decomposes in two exothermic steps that occur at 213.4 and 424.2 °C, with a total mass loss of 70.80%. The corresponding thermograph of **CTAB@TC** consists of four exothermic steps at 58.6–162.4 °C (mass loss 0.79%), 162.4–246.8 °C (mass loss 25.24%), 246.8–339.7 °C (mass loss 63.83%), and 339.7–500.0 °C (mass loss 6.05%). The total mass loss is 95.91% (Figure S2B). The melting points of **SLS@TC** and **CTAB@TC** were obtained by the differential thermal analysis diagrams (DTA). The strong sharp transitions in DTA diagrams suggest that the melting points of **SLS@TC** and **CTAB@TC** are 203–207 and 222–229 °C, respectively (Figure S2), in contrast to the melting point of free **TC** at 191.0 °C (Figure S2C), confirming the purity of the micelles.

##### Differential Scanning Calorimetry (DSC)

**Figure 1:**
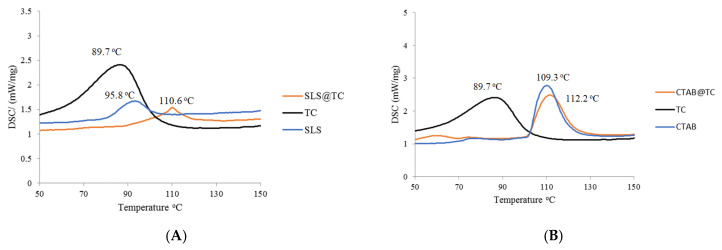
Differential scanning calorimetry (DSC) diagram of **SLS@TC** (**A**) **CTAB@TC** (**B**). TC, tetracycline; SLS, sodium lauryl sulfate; CTAB, cetrimonium bromide.

To confirm whether **TC** interacts with **SLS** or **CTAB** in the solid state to give a composite material or a mixture, DSC studies were carried out. DSC diagrams of **SLS@TC** and **CTAB@TC** are shown in Figure S3. Four endothermic phase transitions at 111.1, 185.8, 229.7, and 259.3 °C are observed in the DSC diagram of **SLS@TC**, while the corresponding ones were observed at 112.3 and 257.6 °C for **CTAB@TC**. The phase of **SLS** at 95.8 °C is observed at 110.6 °C in the corresponding one of **SLS@TC**, suggesting the formation of a composite material (Figure 1). Meanwhile, the transformation at 109.3 °C in the case of CTAB is also observed at 112.2 °C in **CTAB@TC**, indicating that a mixture of the micelle’s components is obtained in this case.

##### Vibrational Spectroscopy

The IR spectra of **TC**, **SLS**, **CTAB**, **SLS@TC**, and **CTAB@TC** are shown in Figures S4 and S5. The IR spectrum of **SLS@TC** is mainly dominated by the vibrations of SLS. Thus, the ν(H-C) vibrations in the IR spectrum of **SLS@TC** are observed at 2918 and 2850 cm^−1^. The vibration band at 1070 cm^−1^ in the spectrum of **SLS@TC** is attributed to ν_asym_ of the S=O, while the vibration at 1403 cm^−1^ in the IR spectrum of **CTAB@TC** is attributed to δ(N^+^(CH_3_)_3_). However, no characteristics bond vibrations such as to ν_asym_ (C=O) or C=C (1637 and 1577 cm^−1^, respectively) of **TC** are observed in the IR spectrum of the micelle owing to the low stoichiometry of **TC**/**SLS** or **TC**/**CTA**B in the micelles.

### 2.3. Solution Studies

#### 2.3.1. UV/vis Study

**Figure 2:**
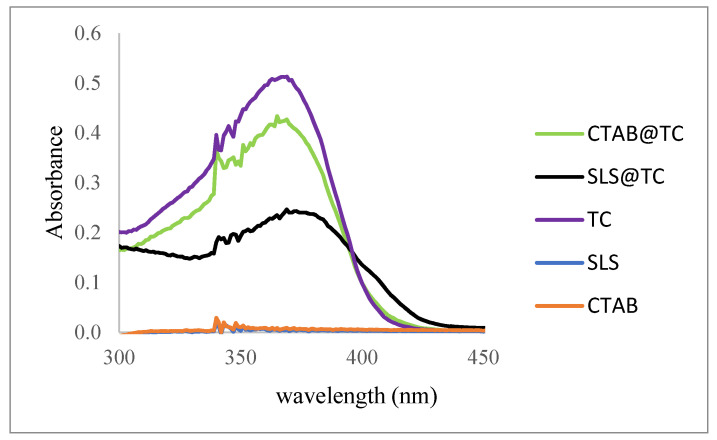
UV/vis spectra of **TC** (10^−4^ M), **SLS** (10^−4^ M), **CTAB** (10^−4^ M), **SLS@TC** (0.18 mg/mL), and **CTAB@TC** (0.29 mg/mL).

The content of **TC** in the micelles was determined by UV/vis spectroscopy. Thus, the UV/vis spectra of five standard solutions in DMSO of **TC** (1 × 10^−4^, 8 × 10^−5^, 6 × 10^−5^, 4 × 10^−5^, 2×10^−5^, 1 × 10^−5^ M) were recorded (Figure S6A). The linear curves of the absorbance at λ_max_ = 369 nm of DMSO solutions of **TC** versus concentration are shown in Figure S6B. Figure 2 shows the UV/vis spectra of **TC**, **SLS**, **CTAB**, **SLS@TC**, and **CTAB@TC**.

Given that both SLS and **CTAB** are UV inactive at 369 nm (Figure 2), any absorption in this wavelength is attributed to **TC**. Therefore, the concentrations of **TC** found in **SLS@TC** and **CTAB@TC** are 8.3% *w/w* (**SLS@TC**) and 7.9% *w/w* (**CTAB@TC**), respectively. These values are in agreement with those found from CMC, namely 8.3% *w/w* (**SLS@TC**) and 7.6% *w/w* (**CTAB@TC**), which correspond to the 17/1 ([**SLS**]/[**TC**]) and 16/1 ([**CTAB**]/[**TC**]) molar ratios, respectively. The MIC, MBC, biofilm elimination concentration (BEC), and so on for the micelles **SLS@TC** and **CTAB@TC** were determined with respect to **TC** using its concentrations in micelles, as these were derived by UV/vis spectroscopy. The concentrations of micelles in solutions, however, are far higher to their CMCs, which ensures their stability.

#### 2.3.2. ^1^H NMR Study

**Figure 3:**
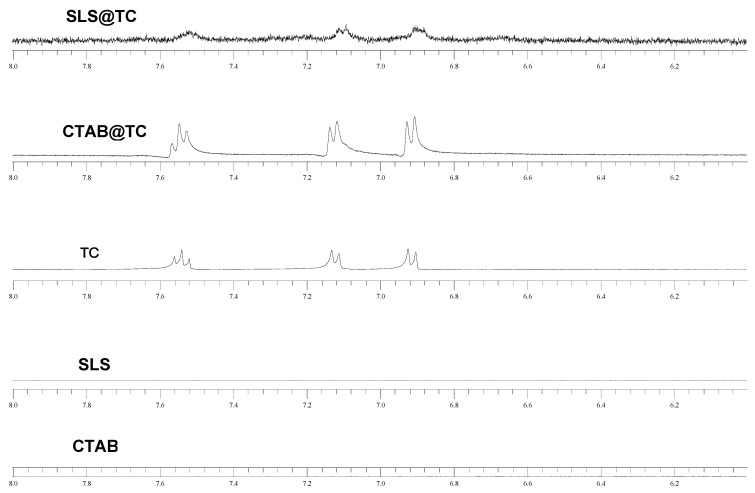
Nucleus magnetic resonance (NMR) spectra (8–6 ppm) in DMSO-*d*_6_ of **SLS@TC**, **CTAB@TC**, **TC**, **SLS**, and **CTAB**.

The ^1^H NMR spectra in DMSO-*d*_6_ of **TC**, **SLS**, **CTAB**, **SLS@TC**, and **CTAB@TC** (8–6 ppm) are shown in Figure 3. The resonances signals at 7.56–7.52 (t) ppm in the spectrum of **TC** are assigned to the amide H[NH_2−_] group (Scheme 1), while the signals at 7.13–7.10 (d) ppm and 6.92–6.90 (d) ppm are attributed to the aromatic protons HC_arom_^2–4^ of **TC** (Scheme 1) [38]. The presence of these signals in the micelles spectra **SLS@TC** (7.52 (br), 7.12–7.10 (d), and 6.91–6.87 (d) ppm) and **CTAB@TC** (7.56–7.53 (t), 7.14–7.12 (d), and 6.93–6.91 (d) ppm) confirms the encapsulation of the antibiotic in the micelles.

### 2.4. Probing Morphological Alterations by Means of Ultrasonically-Induced Transient Birefringence Experiments and ATR Spectroscopy

#### 2.4.1. Transient Acoustically-Induced Birefringence Measurements

**Figure 4:**
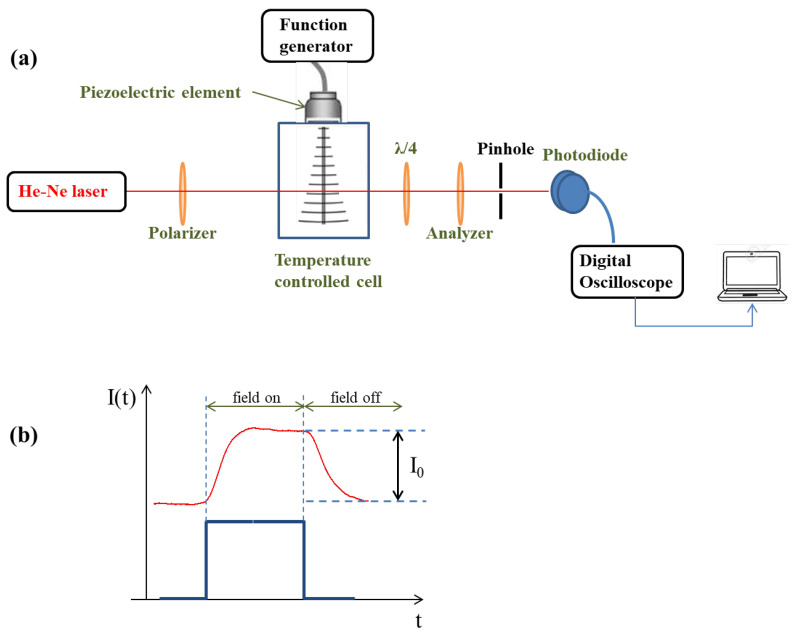
(**a**) Experimental setup for acoustically-induced optical birefringence measurements. See text for a detailed description. (**b**) The applied ultrasonic square-pulse and the birefringence trace when the acoustic field is on and off.

For measuring the transient acoustically-induced birefringence, we used an instrument depicted in Figure 4. Its components have been described in detail elsewhere [39]. A solution of the sample was loaded in a cylindrically-shaped and thermostated non-birefringent glass cell with 1 cm optical path-length. A square electric pulse was delivered to a piezoelectric element, which further transmitted the ultrasonic wave into the liquid in perpendicular direction with respect to the light propagation. A 5 mW linearly polarized He-Ne laser (Melles-Griot, λ = 632.8 nm) working at TEM00 was used as the probe light source. In order to perform birefringence measurements, the cell was placed between a polarizer oriented at π/4 and a quarter wave plate (λ/4-plate) in cross-polarization relative to the polarizer. The quarter wave plate was followed by an analyzer with this plane rotated by an angle *α* clockwise or counterclockwise relative to the λ/4-plate. The transmitted intensity of the laser beam was detected by a fast photodiode and monitored in a digital oscilloscope.

When birefringence is induced by ultrasound in the liquid sample, a change in the transmitted intensity Δ*I_δ_(t)* = *I_δ_(t)* − *I_α_* is observed. *Ι_δ_(t)* is the light intensity attributed to phase retardation *δ* and *I_α_* corresponds to the light intensity at an angle *α* when the acoustic field is off. The intensity change is related to the induced optical retardation *δ(t)* through the following equation:

(1) $$\frac{\Delta I_{\delta }(t)}{I_{\alpha }}=\frac{\sin^{2}\left(a+\frac{\delta }{2}\right)-\sin^{2}a}{\sin^{2}\alpha }$$

**Figure 5:**
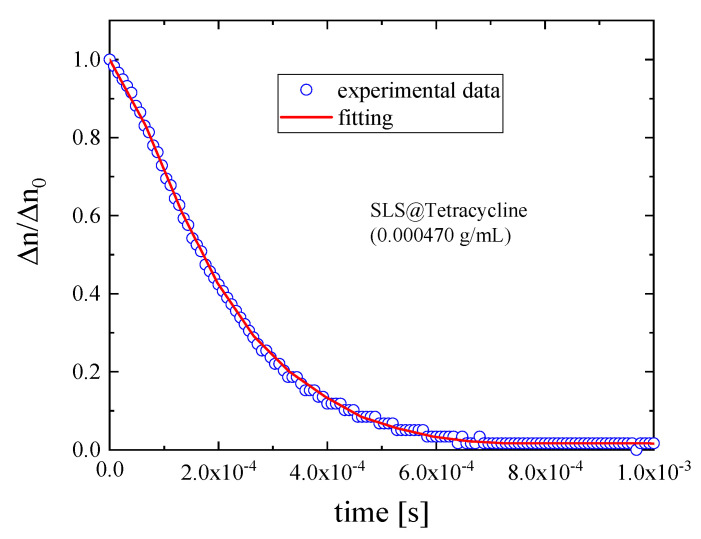
Representative fitting example of the birefringence decay recorded for the **SLS@TC** solution with concentration 0.000470 g/mL. The ultrasound frequency was 774 kHz. Open circles: experimental data (only one-third of the experimental points are shown for clarity); thick solid line: total fit curve.

The induced birefringence is associated with the retardation through the following:

(2) $$\Delta n=\frac{\delta \lambda }{2\pi d}$$

where *d* is the optical path-length and *λ* is the laser wavelength. The applied ultrasonic pulse and the acoustically-induced birefringence are presented in Figure 4b. When the acoustic square pulse perturbation is applied, the measured light intensity exhibits two different relaxations after switching the field on and off. During the acoustic field-on regime, the rise of the birefringence is given by the following:

(3) $$\Delta n(t)=\Delta n_{\max}\left(1-e^{-{\left(t/\tau \right)}^{\beta }}\right)$$

while, after sudden termination of the field, the birefringence decay is as follows:

(4) $$\Delta n(t)=\Delta n_{\max}\;e^{-{\left(t/\tau \right)}^{\beta }}$$

where Δ*n_max_* corresponds to the steady state birefringence when the acoustic field is on, *τ* is the characteristic relaxation time for the rise and decay of the birefringence signal, and *β* is assigned to the width of the relaxation time distribution. A simple exponential function (*β* = 1) is inadequate to describe the experimental data, implying a distribution of the relaxation times. The smaller the value of *β*, the larger the distribution of the relaxation times. A representative fitting example of the normalized birefringence decay Δ*n/*Δ*n_max_* is presented in Figure 5, where an acceptable goodness of fit is observed.

For all measurements, the liquid sample was kept in a sealed cuvette under isothermal conditions corresponding to 37 °C. The entire volume of the liquid was exposed uniformly to the applied acoustic field. As no flow is permitted in and out of the cell, possible directional flow or liquid sharing is missing.

The application of the acoustic pulse allows us to observe the molecular motion dynamics directly in the time-domain by monitoring the induced birefringence trace. In general, the acoustic field will induce several types of dynamic processes in isotropic liquids and solutions at equilibrium involving intermolecular interactions. As the acoustic field is switched on, molecules are forced to orient along the propagation of the ultrasound and the whole system becomes un-axial and birefringent. After switching off the acoustic field, the induced orientation will fade away through a diffusion process of the molecules. The rotation is strongly influenced by the polarization of the neighboring molecules through dipole–induced-dipole interactions (DID). The strength of these interactions is subject to the distance and relative position between molecules. After the system is not exposed to the acoustic field, the DID interactions will level off to a minimum free-energy state, while molecules will continue to rotate because of their inertia, but under the restrictions posed by the neighboring molecules. These constraints induce molecular libration relaxation on a femtosecond timescale, which is ultrafast and beyond the limits of the ultrasonically-induced birefringence technique. Molecular liquids and their mixtures exhibit usually fast birefringence response (see, e.g., [39] and references therein). In the case of **SLS@TC** and **CTAB@TC** dilute solutions, a relatively slower response is observed because of the peculiar nature of the underlying dynamics, where a self-organization into larger supramolecular aggregates takes place, leading to a dynamically complex structure. Thus, both **SLS@TC** and **CTAB@TC** solutions cannot be considered as a dispersion of anisotropic particles in a liquid matrix with no polarization and steric effects.

**Figure 6:**
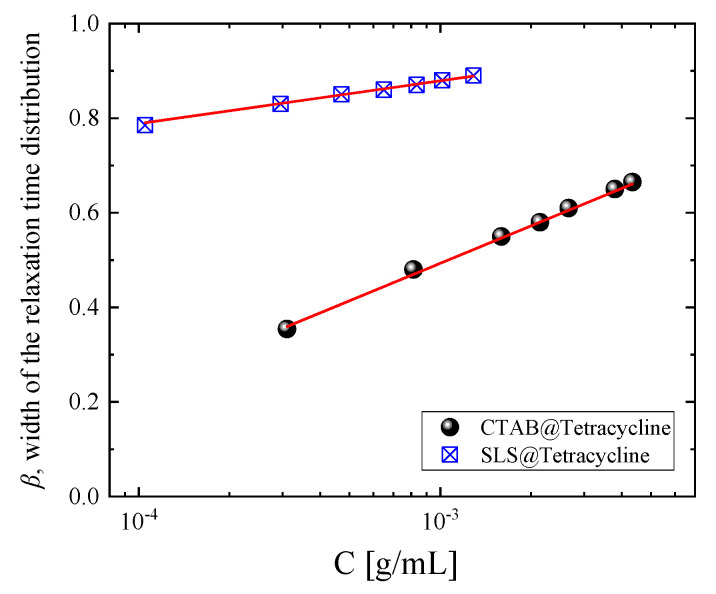
Concentration dependence of *α* parameter attributed to the width of the relaxation time distribution for **SLS@TC** and **CTAB@TC** systems. All measurements correspond to 37 °C. See text for details concerning the fitting procedure.

The experimental results recorded in this work were adequately fitted by a stretched exponential function (Equation (4)), exhibiting analogous behavior with other complex liquids [40,41]. With switching off the acoustic field, the recorded birefringence trace decreases to zero as a result of thermal orientational fluctuations (Brownian rotation) of the particles. The corresponding relaxation time *τ* is related to the hydrodynamic volume *V_h_* of the particle through the following equation [42]:

(5) $$\tau =\frac{\eta V_{h}}{k_{B}T}$$

where *T* is the absolute temperature, *k_B_* is the Boltzmann’s constant, and *η* is the shear viscosity of the solvent. The relaxation times from the birefringence measurements can be estimated from the fitting procedure and used to calculate the hydrodynamic volume of the entities in the solutions. In Figure 6, the concentration dependence of the fitting parameter *α* is presented for both **SLS@TC** and **CTAB@TC** systems. It seems that the relaxation time distribution of **CTAB@TC** solutions is wider compared with the relaxation time distribution of **SLS@TC** solutions. Furthermore, for each system, the distribution width becomes narrow monotonically from dilute to dense solutions.

**Figure 7:**
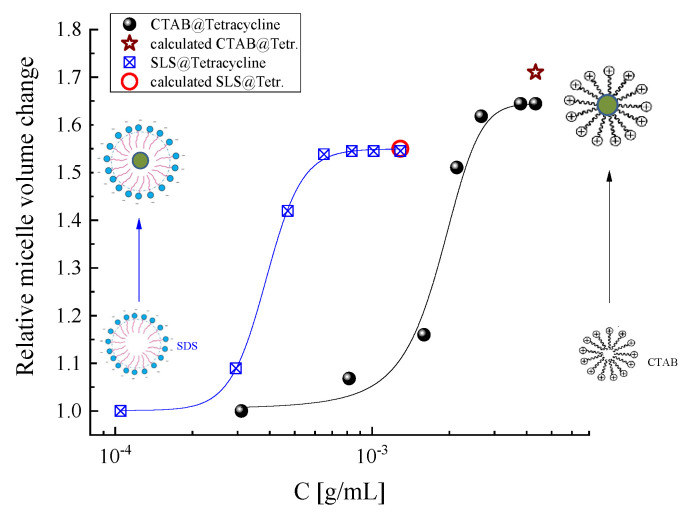
Relative micelle volume change as a function of concentration for **SLS@TC** (squares) and **CTAB@TC** (solid circles) solutions. The open circle and star symbols correspond to the theoretically calculated volume change for **SLS@TC** and **CTAB@TC**, respectively. Lines are guides to the eye. A schematic representation of the initial and final micelle is also shown.

The hydrodynamic volumes, as obtained from Equation (5), were used to confirm the incorporation of tetracycline in **SLS** and **CTAB** micelles and to quantitatively follow the volume change. The relative micelle volume changes are shown in Figure 7. The results clearly indicate the incorporation of **TC** in the parental micelles for both surfactants. We also calculated the theoretically predicted volume change between the initial and final complex micelle. We considered the diameter of the initial **SLS** micelle as approximately two times the size of the **SLS** (19.5 Å) and the diameter of the initial **CTAB** micelle as two times the size of the **CTAB** (24.2 Å), respectively. The diameter of the **SLS@TC** and **CTAB@TC**, after the incorporation of **TC** in the parental micelle, is calculated as the sum of the parental micelle diameter plus the mean diameter of the **TC** molecule (45.11 Å). The calculation of the mean **TC** diameter was carried out in vacuum (without any interactions), with the use of the Gaussian 09 series of programs [43]. The theoretically calculated volume changes for **SLS@TC** and **CTAB@TC** micelles are in agreement with the experimental data, confirming the proposed underlying mechanism.

Additional information concerning the length distribution of the micelles can be obtained from the birefringence decay measurements. In the context of the Watson–Jennings approach, the length distribution is assumed to follow a log-normal behavior and is given by the following [44]:

(6) $$P(d)=\frac{1}{d\sigma \sqrt{2\pi }}\exp\left\{-\frac{{\left[\ln\left(d/d_{m}\right)\right]}^{2}}{2\sigma^{2}}\right\}$$

The above equation is a two-parameter equation, where *d_m_* is the median and *σ* corresponds to the breadth of the size distribution. Both parameters can be estimated as the solution of the following system of equations:

(7) $$D_{WJ}=\lim_{t\to 0}\left\{\frac{d}{dt}\left(\ln\frac{\Delta n(t)}{\Delta n_{0}}\right)\right\}=-\frac{6d_{m}^{3}}{F_{\theta }}\exp\left(15\sigma^{2}/2\right)$$

(8) $$I_{WJ}=\int_{0}^{\infty }\frac{\Delta n(t)}{\Delta n_{0}}dt=\frac{\exp\left(9\sigma^{2}\right)}{D_{WJ}}$$

**Figure 8:**
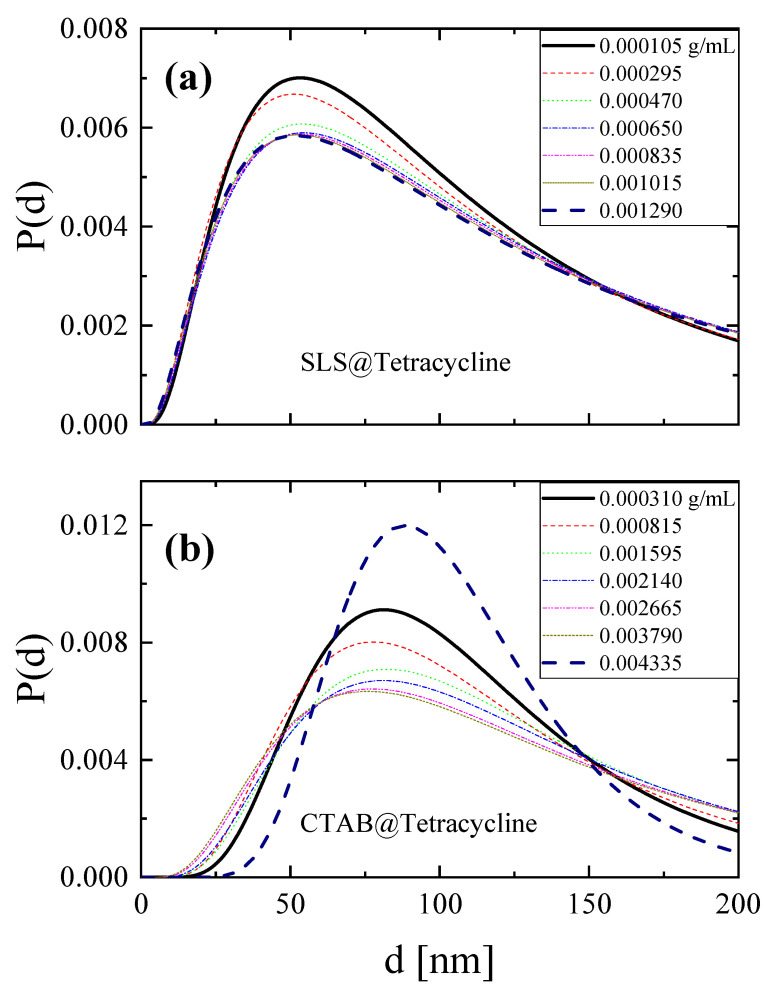
Distribution of hydrodynamic diameters of the **SLS@TC** (**a**) and **CTAB@TC** (**b**) micelles.

The *D_WJ_* factor is the initial logarithmic derivative, while *I_WJ_* represents the area under the birefringence trace. Following the above methodology, we estimated the particle size distributions for both systems and the results are presented in Figure 8. In both systems, the size distribution is shifted and widened with increasing solution concentration, with the **CTAB@TC** system exhibiting the most intense changes. The different behavior in terms of particle aggregation arises from the different head group properties, which in turn influence the extent of the hydrophobic or hydrophilic tail interactions. Both **SLS** and **CTAB** form complexes that involve an arrangement of electrostatic and hydrophobic interactions. The shift in the distribution functions is related to the characteristic particle size obtained from the birefringence relaxation due to rotational diffusion.

**Figure 9:**
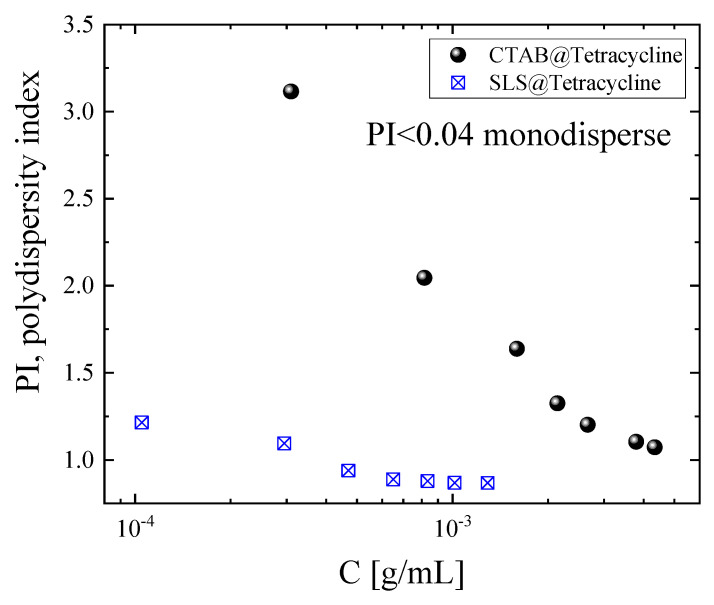
Polydispersity index (PI) for **SLS@TC** and **CTAB@TC** systems as a function of concentration.

The relative variance of the distribution for each case can be evaluated from the polydispersity index (PI) considering the standard deviation and the mean size of the distribution, and the results are presented in Figure 9. The lower the PI values, the lower the dispersion and, below 0.04, the system can be considered as monodispersed. It seems that both systems are polydispersed, with **CTAB@TC** more dispersed than the **SLS@TC**. The size dispersion behaves in an analogous manner with relaxation time dispersion, as observed in Figure 6. The analysis of the ultrasonically-induced birefringence relaxation permits to comprehend the distribution of the hydrodynamic diameter or volume of the micelles formed. Furthermore, on the basis of the concentration-dependent birefringence measurements, it is possible to distinguish between simple and **TC** incorporated micelles and obtain a more quantitative description of the mechanism efficiency. The relaxation time and hydrodynamic diameter distribution highlight the complexity of the interactions involved in the intermolecular assemblies present in surfactant-based solutions.

#### 2.4.2. Attenuated Total Reflection Measurements

The infrared spectra of the solutions in attenuated total reflection (ATR) mode were recorded by employing an Alpha spectrometer (Bruker) in the 500–4000 cm^−1^ spectral region with a Deuterated Triglycine Sulfate (DTGS) detector. The infrared radiation is transferred to the ATR crystal made of ZnSe with an angle of incidence set at 45° and all spectra were recorded with a 2 cm^−1^ spectral resolution. The ATR crystal surface was completely covered by the liquid sample, thus permitting quantitative estimations from the spectra. The ATR crystal was cleaned with isopropanol after each spectrum acquisition to ensure that no impurities from previous measurements were retained on the crystal’s surface.

#### 2.4.3. Vibrational Modes—Short-Range Structure

**Figure 10:**
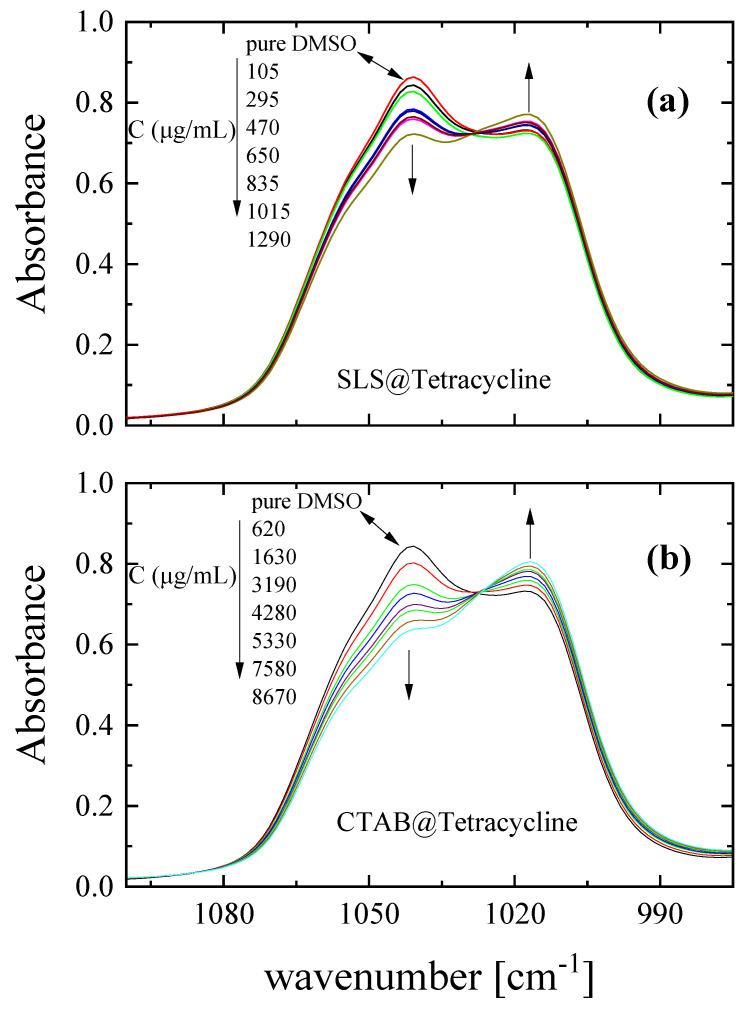
Attenuated total reflection (ATR) spectra in the spectral region of the S-O symmetric (1018 cm^−1^) and antisymmetric (1042 cm^−1^) stretching of DMSO dimers as a function of concentration for **SLS@TC** (**a**) and **CTAB@TC** (**b**) solutions. The two bands at 1041 and 1055 cm^−1^ are associated to self-associated and un-associated DMSO molecules, respectively. The former species are incorporated in chain- and cyclic-like structures [45,46,47].

The spectral fingerprints of the DMSO solvent are expected to dominate the spectra of all solutions in such low concentrations. Indeed, the main absorption observed in Figure 10 is ascribed to the S-O symmetric (1018 cm^−1^) and antisymmetric (1042 cm^−1^) stretching vibration of DMSO dimers [45,46,47]. Nevertheless, the spectral variations observed in Figure 10 are attributed to changes in the local environment due to surfactant self-association and to the replacement of the dipole–dipole interactions between solvent molecules (DMSO) with hydrogen-bonded “new” species with DMSO dimers. This interplay is observed as an isosbestic point in the concentration-dependent spectra of both **SLS@TC** and **CTAB@TC** systems presented in Figure 10.

The spectrum of **SLS** reveals a set of antisymmetric and symmetric stretching vibrations of -CH_2_- and -CH_3_ groups of the hydrocarbon tail in the 3000–2800 cm^−1^ range [48,49]. The bending modes of these groups are observed at ~1461 and ~1377 cm^−1^, respectively. The two peaks located at 1237 and 1200 cm^−1^ are assigned to antisymmetric S-O stretching, while the bands at 1060 and 974 cm^−1^ are assigned to symmetric S-O stretching vibration of the -OSO_3_^−^ species [48,49]. The spectrum of the isolated sulfate SO_4_^2−^ species with tetrahedral symmetry (T_d_) exhibits a totally symmetric S-O stretching *ν_1_* mode at 980 cm^−1^, which is IR inactive, and a triply-degenerate antisymmetric *ν_3_* band located at 1102 cm^−1^ [50]. In the case of the **SLS** system, the sulfonate head-group reveals lower tetrahedral symmetry with the C_3V_ point group instead of T_d_. Moderate distortion of the tetrahedral units results in a splitting of the antisymmetric stretching mode into a double-degenerate and a non-degenerate band observed at high- and low-frequencies, respectively [51,52]. Additional distortion causes further reduction of the tetrahedral symmetry to the C_2V_ point group with a subsequent splitting of the *ν_3_* band into three counterparts in the frequency range from 1250 to 1050 cm^−1^. The correlation diagram presented in Table 1 shows the band splitting into a large number of bands after moderate and severe distortions of the tetrahedral units with initial T_d_ symmetry.

An interesting spectral observation in the **SLS@TC** system is that the antisymmetric stretching band of SLS at ~1208 cm^−1^, assigned to the charged ionic sulfonate head OSO_3_^−^, is shifted in frequency above a concentration of 295 μg/mL. This finding is attributed to the gradual hydrogen bond formation between one or two sulfonate oxygen atoms with the tetracycline’s functional groups, indicating the transition from SLS-micelle to complex **SLS@TC** micelle. The non-degenerate A_1_ antisymmetric stretching vibration is parallel to the direction of the SLS molecule, and thus is sensitive to the presence of tetracycline molecule, while the doubly-degenerate E vibration is perpendicular to the direction of the **SLS** molecule and is prone to lateral interaction between surfactant–surfactant entities [52].

**Figure 11:**
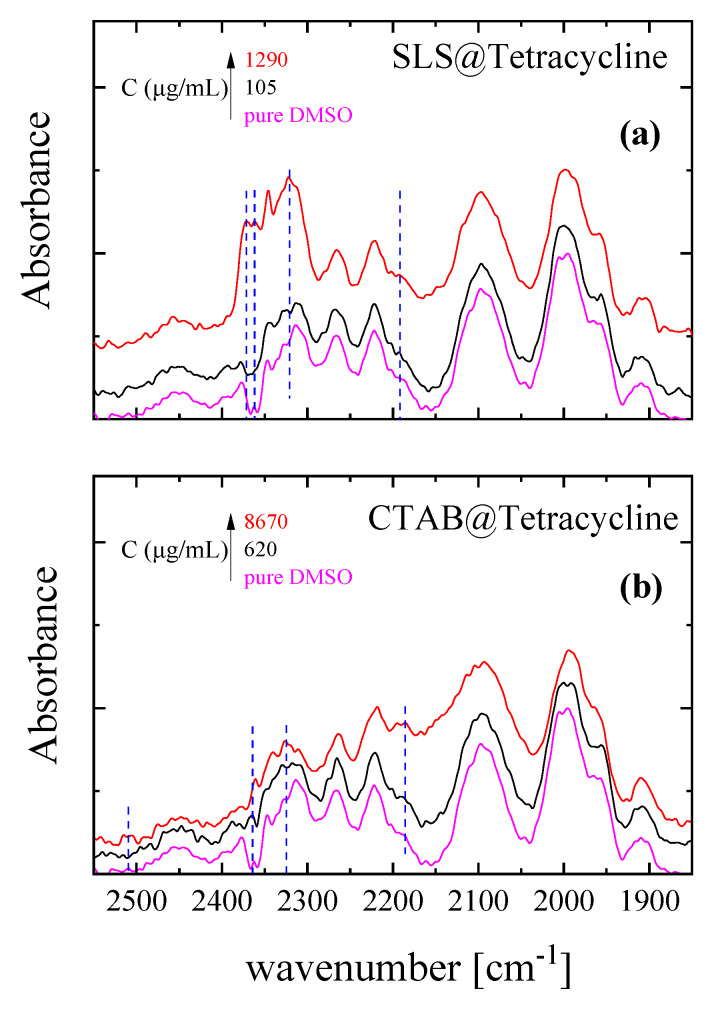
ATR spectra in the [2550–1850] spectral region for **SLS@TC** (**a**) and **CTAB@TC** (**b**) solutions for selected concentrations. The spectrum of pure solvent is also shown for comparison. Dotted lines indicate intensity variations attributed to micelle formation.

Relative intensity changes and new bands are observed in the ATR spectra of solutions with increasing solution concentration in addition to the bands attributed to the main functional groups of the surfactant (**SLS**, **CTAB**), tetracycline, and the solvent (DMSO). These spectral observations are denoted with dotted lines in Figure 11 for **SLS@TC** and **CTAB@TC** solutions for selected concentrations. The spectrum of DMSO solvent is also shown in the same spectral region for comparison. These observations account for the above described interactions related to complex micelle formation.

### 2.5. Antibacterial Studies

#### 2.5.1. Effect of **SLS@TC**, **CTAB@TC**, **TC**, **SLS**, and **CTAB** on the Growth of Microbial Strains

The antimicrobial potency of **SLS@TC**, **CTAB@TC**, **TC**, **SLS**, and **CTAB** was tested against Gram negative (*P. aeruginosa* and *E. coli*) and Gram positive (*S. epidermidis* and *S. aureus*) bacterial strains by means of MIC (Table 2, Figures S7–S10). MIC is defined as the lowest concentration needed for the inhibition of the bacterial growth [21,22,23,24,25,26,27,28]. The **TC** is suspended in water for the experiments. The ranges of MIC values of **SLS@TC** and **CTAB@TC** are 3.7–27.2 μΜ and 0.07–5.8 μΜ, respectively. The biological effects of the micelles **SLS@TC** and **CTAB@TC** against the strains tested were determined with respect to **TC** using its concentrations in the micelles, derived by UV/vis spectroscopy, that is, 8.3% *w/w* **TC** in **SLS@TC** and 7.9% *w/w* **TC** in **CTAB@TC**. Thus, the concentrations of micelles in solutions remain higher their CMCs, reserving their stability, but lower than the MIC values of the surfactants themselves.

**SLS@TC** and **CTAB@TC** show a higher antibacterial effect than **TC** towards the Gram negative and Gram positive bacteria. This is attributed to their water solubility, which enhances their bioavailability, in contrast to the water insoluble **TC**. Both micelles exhibit the highest activity towards the bacterial strains of *S. epidermidis* and *S. aureus*, in contrast to **TC**. **SLS@TC** and **CTAB@TC** exhibit up to 6-fold and up to 540-fold stronger activity than **TC** towards *S. epidermidis*, respectively. The MIC values of **SLS** and **CTAB** range from 0.4 to >250 μΜ and they exhibit lower activity than the micelles of tetracycline. However, **CTAB** exhibits higher activity than **SLS** against all tested strains, which is also associated with the higher activity of **CTAB@TC** than **SLS@TC**. Especially, **CTAB@TC** is up to 89-fold more active than **SLS@TC** against S*. epidermidis*.

#### 2.5.2. Evaluation of the Minimum Bactericidal Concentration (MBC)

The MBC value is defined as the lowest concentration of an antibacterial agent that can eliminate 99.9% of the bacterial inoculum [21,22,23,24,25,26,27,28]. The MBC values of **SLS@TC**, **CTAB@TC**, **TC**, **SLS**, and **CTAB** were determined against *P. aeruginosa*, *E. coli*, *S. epidermidis* and *S. aureus* (Figures S11–S14). The MBC values of **SLS@TC** and **CTAB@TC** range from 9.1–116.0 and 0.5–95.0 μΜ, towards all the bacteria, respectively (Table 2). The MBC/MIC ratio classifies antibacterial agents into bactericidal or bacteriostatic ones. Thus, (i) bactericidal agents exhibit MBC/MIC lower than 2, while (ii) when this ratio is higher than 4, the agent is classified as bacteriostatic. Therefore, both micelles are bacteriostatic agents towards *P. aueroginosa*, *E. coli*, *S. epidermidis* and *S. aureus*. However, in the case of *S. epidermidis*, the **SLS@TC** is a bactericidal agent against (MBC/MIC = 1.9 (Table 2)).

#### 2.5.3. Determination of the Inhibition Zone (IZ) through the Agar Disk-Diffusion Method

**Figure 12:**
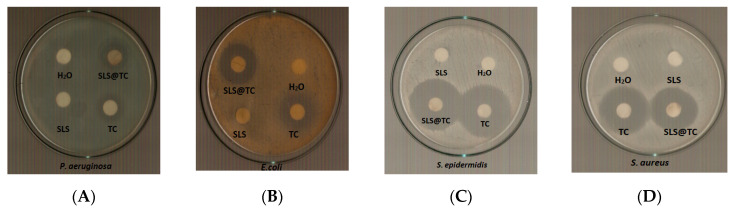
Inhibition zones of **SLS@TC** towards *P. aeruginosa* (**A**), *E. coli* (**B**), *S. epidermidis* (**C**), and *S. aureus* (**D**).

**Figure 13:**
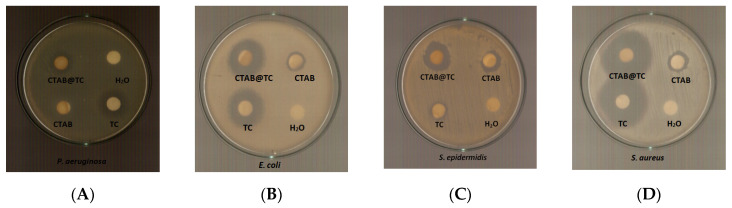
Inhibition zone of **CTAB@TC** towards *P. aeruginosa* (**A**), *E. coli* (**B**), *S. epidermidis* (**C**), and *S. aureus* (**D**).

The agar disk-diffusion method was used in order to survey the sensitivity of *P. aeruginosa*, *E.coli*, *S. epidermidis*, and *S. aureus* towards **SLS@TC**, **CTAB@TC**, **TC**, **SLS**, and **CTAB** at the concentration of 1 mM with regards to **TC** [21,22,23,24,25,26,27,28]. Therefore, the solutions of 1 mM **SLS@TC**, **CTAB@TC**, and **TC** contain the same amount of **TC** (1 mM), which makes their biological effect comparable. The diameter of inhibition zones of bacterial growth was measured after 20 h (Table 2, Figure 12 and Figure 13). Given that the solutions of **SLS@TC** and **CTAB@TC** contain **TC** in the same concentration, their IZs towards the tested bacteria lie between 31.8–23.8 and 13.7–34.0 mm, respectively, which are longer than the corresponding ones of **TC** itself. Although **CTAB@TC** forms IZs comparable with **TC**, **SLS@TC** creates significantly greater zones. As both **SLS@TC** and **CTAB@CT** are water soluble, this effect should be attributed to the formation of the composite material, confirming the conclusion of MBC (see above).

Given that the CMCs of the micelles of **TC** with SLS and CTAB are 1:17 (**SLS@TC**) and 1:16 (**CTAB@TC**), the concentration of the surfactants in the case of IZ’s measurements could be 17 and 16 mM, respectively, as the corresponding one of **TC** is 1 mM. However, micelles are considered as single entities, which build up as a result of hydrophobicity of the surfactants at higher than their CM concentrations. They can also encircle small molecules such as **TC** in one entity. Thus, **SLS@TC** and **CTAB@TC** are single entities and their concentrations are equal to the corresponding one of **TC** (1 mM), which is the limiting ingredient in micelles. Consequently, the same concentration (1 mM) was used for **SLS** or **CTAB** during the IZ’s measurement. While **SLS** and **CTAB** show negligible or no antibacterial activities at the concentration of 1 mM, their micelles SLS@TC and **CTAB@TC** do. Moreover, as both **SLS** and **CTAB** are already used for drug delivery [30,31,32,33,34], the **CTAB** cation is an effective antiseptic agent against bacteria and fungi [35]; the **SLS** is an anionic body detergents-cleansers (shower gels and toothpastes) ingredient, without any harmful irritations [36], which has been recognized by USFDA as a harmless food ingredient (21 CFR 172,822); the antimicrobial activities of **SLS** and **CTAB** are expected to be limited.

Moreover, microbial strains to which an antimicrobial agent causes IZ ≥ 17 mm are considered as susceptible to it: (ii) those with 13 ≤ IZ ≤ 16 mm are intermediate, while (iii) those with IZ ≤ 12 mm, are considered as resistant strains against the agent [21,22,23,24,25,26,27,28]. The gram positive and negative strains used here are susceptible to **SLS@TC** and **CTAB@TC** (Table 2). However, *P aeruginosa* shows intermediate sensitivity towards **CTAB@TC**.

#### 2.5.4. Effect on Biofilm Formation by **SLS@TC** and **CTAB@TC**

Nowadays, 80% of the clinical infections are biofilm related. Especially, the ophthalmic biofilm infections are difficult to treat. Biofilm elimination can be achieved by applying metalo-antibiotic or their micelles [24,25,26,27].

The effect of **SLS@TC**, **CTAB@TC**, and **TC** against biofilms formed by *P. aeruginosa* and *S. aureus* was evaluated by the biofilm elimination concentration (ΒΕC) using crystal violet assay [24,25,26,27]. The ΒΕC is defined as the required concentration to achieve at least a 99.9% reduction in the viability of biofilm bacteria. The BEC values of **SLS@TC**, **CTAB@TC**, and **TC** are 557, 1875, and 427 μΜ, respectively, against *P. aeruginosa*, while they are 1913, 983, and >2300 μΜ [53], respectively, against *S. aureus* (Figures S15–S17). Thus, **SLS@TC** and **CTAB@TC** are more efficient against the biofilm of *S. aureus*, while they are less towards *P. aeruginosa* than free **TC**.

#### 2.5.5. Evaluation of In Vitro Toxicity

The in vitro toxicity of **SLS@TC**, **CTAB@TC**, **TC**, **SLS**, and **CTAB** was evaluated against normal human corneal epithelial cells (HCECs) by sulforhodamine B (SRB) assay. The IC_50_ values were determined after incubation of cells with the agents for a period of 48 h. The IC_50_ values of **SLS@TC**, **CTAB@TC**, **TC**, **SLS**, and **CTAB** are 14.1, 0.09, >60, >60, and 3.7 μΜ, respectively (Table 2). The synthesized micelles are more toxic than **TC**.

#### 2.5.6. Evaluation of In Vitro Genotoxicity

**Figure 14:**
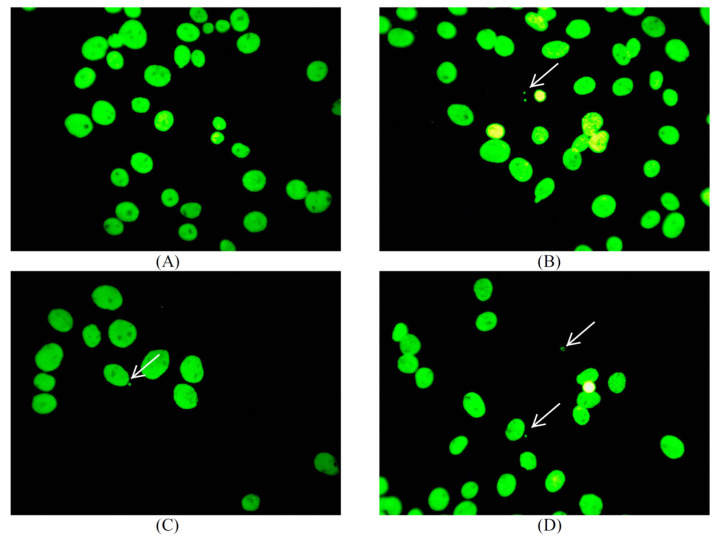
Representative snapshots of micronucleus formed in untreated HCECs (**A**) and upon their treatment with **SLS@TC** (**B**), **CTAB@TC** (**C**), and **TC** (**D**) for 48 h.

The micronucleus assay was employed for the evaluation of in vitro genotoxicity. It is widely used to monitor genetic damage in the cells and in order to evaluate the mutagenic, genotoxic, or teratogenic effect of metallodrugs [54]. The in vitro genotoxicity of **SLS@TC**, **CTAB@TC**, and **TC** was tested towards HCECs at their IC_50_ values (Figure 14). The corresponding frequency of micronucleus after treatment of HCECs upon their treatment with **SLS@TC**, **CTAB@TC**, and **TC** is (2.0 ± 0.2), (1.9 ± 0.7), and (2.5 ± 0.7)%, respectively, in contrast to (2.4 ± 0.2)% for non-treated cells. The micronucleus frequencies of the micelles and **TC** indicate their non-mutagenic or genototic activity towards HCECs.

### 2.6. In Vivo Toxicity Evaluation, by Brine Shrimp Artemia Salina

The in vivo toxicity of the **SLS@TC** and **CTAB@TC** was screened by brine shrimp *Artemia salina* assay. The brine shrimp *Artemia salina* lethality test is a preliminary toxicity test, useful in predicting biological activities such as cytotoxic, phototoxic, and pesticidal activities [28,55].

The % survival of *Artemia salina* larvae in increasing concentrations of solutions with or without **SLS@TC** and **CTAB@TC** after 24 h is summarized in Table 3. The lethality was noted in terms of deaths of larvae. The mortality rate of *brine shrimp* larvae was found upon their incubation with **SLS@TC** and **CTAB@TC** in concentrations equal to MIC^min^, MIC^max^, 2 × MIC^max^, and 3 × MIC^max^ against *P. aeruginosa*, *E. coli*, *S. epidermidis*, or *S. aureus* respectively, suggesting low toxicity at MIC^max^ (Table 3).

## 3. Conclusions

The disadvantage of the negligible water solubility of tetracycline (**TC**), a well-known antibiotic of clinical use, is overcome by the development of its micelles with formulae **SLS@TC** and **CTAB@TC**. Both micelles were characterized by spectroscopy techniques and ultrasonic imaging. The hydrodynamic volumes are obtained from transient acoustically-induced birefringence measurements. The results indicate the incorporation of **TC** in the parental micelles for both surfactants. While the diameters of **SLS** or **CTAB** micelles are twice the corresponding ones of **SLS** or **CTAB**, respectively, the diameters of the **SLS@TC** and **CTAB@TC** are the sum of the diameters of the parental micelles and that of tetracycline itself.

**SLS@TC** and **CTAB@TC** show a higher antibacterial effect than **TC** towards the Gram negative and Gram positive bacteria, because of their water solubility and their bioavailability in contrast to insoluble **TC**. Especially, **SLS@TC** and **CTAB@TC** exhibit up to 6-fold and up to 540-fold stronger activity than free **TC** towards *S. epidermidis*, respectively. Both micelles are bacteriostatic agents towards the tested strains. Moreover, **SLS@TC** and **CTAB@TC** exhibit greater inhibitory efficiency against the biofilm of *S. aureus* than **TC** itself, while they are less towards the corresponding one from *P.* *aeruginosa*. The in vitro genotoxic studies show that the micronucleus frequencies indicate the non-mutagenic or genotoxic activity of micelles towards HCECs. The in vivo toxicity studies towards *brine shrimp* larvae suggest low toxicity at MIC^max^ for both micelles. The irritating side effect that both agents might be caused by the presences of SLS or CTAB is overcome because of their low effective concentration (MIC_max_ < 120 μΜ or 0.01% *w/w*), which is lower than the irritant one (<0.1% *w/w*) [36]. Therefore, **SLS@TC** and **CTAB@TC** can be candidates for the development of new antibiotics.

## Experimental

*Materials and instruments:* All solvents used were of reagent grade. **TC** (98.0–102.0% (HPLC)) was purchased from SIGMA St. Louis, MO, USA product of China. **SLS** (Sigma–Adrich St. Louis, MO, USA) and **CTAB** (Merk India (MITC), Tower 3 Bengaluru, Karnataka, India) were used with no further purification. Tryptone and soy peptone were purchased from Biolife. Agar was purchased from Sigma-Aldrich St. Louis, MO, USA product of Spain. Sodium cloride, D(+)-Glucose, and di Potassium hydrogen phosphate trihydrate were purchased from Merck. Melting points were measured in open tubes with a Stuart Scientific apparatus and uncorrected IR spectra in the region of 4000–370 cm^−1^ were obtained using a Cary 670 FTIR spectrometer, Agilent Technologies. The ^1^H-NMR spectra were recorded on a Bruker AC 400 MHz FT-NMR instrument in DMSO-d*_6_* solution. A UV-1600 PC series spectrophotometer of VWR was used to obtain electronic absorption spectra. Brine Shrimp Eggs (*Artemia salina*) were purchased from Ocean Nutrition.

*Synthesis of **SLS@TC** and **CTAB@TC**:* 0.222 g (0.5 mmol) of tetracycline in 15 mL methanol was mixed with 2.448 g (8.5 mmol) SLS or 2.915 g (8.0 mmol) CTAB in 15 mL in dd water solution. The molar ratios for [SLS]/[TC] and [CTAB]/[CT] were 16/1 and 17/1, respectively, equal to their CMC values. The solution was stirred for 3 h and concentrated to dryness using a rotary evaporator. The oily remains were re-dissolved in 10 mL of diethyl ether and the solution was filtered off. The pure brownish solid micelles were obtained from slow evaporation of the clear ether solution.

***SLS@TC***: yield 30%; **TC** content in **SLS@TC**: 5.22 ± 1.68% *w/w*; melting point: 204–207 °C (change of colour); IR (cm^−1^): 2918 vs, 2851 s, 1608 m, 1468 m, 1216 s, 1080 s, 823 s, 722 m, 631 w, 586 w, 421 m; ^1^H-NMR (ppm) in DMSO-d*_6_*: 7.53–7.50 (t), 7.12–7.09 (d), 6.90–6.87 (d), 3.69–3.66 (t), 2.51 (s), 1.49–1.46 (t), 1.25 (s), 0.87–0.84 (t) ; UV/vis (DMSO): λ_max_ = 271, 369 nm.

***CTAB@TC***: yield 25%; **TC** content in **CTAB@TC**: 4.96 ± 0.16% *w/w*; melting point 237–243 °C (change of colour); IR (cm^−1^): 3015 w, 1463 s, 1402 m, 1247 m, 1205 s, 1079 m, 962 s, 911 m, 829 m, 718 s, 626 m, 591 s; ^1^H-NMR (ppm) in DMSO-d_6_: 7.51–7.52 (t), 7.13–7.11 (d), 6.92–6.90 (d), 3.05 (s), 2,51 (s), 2.41 (s), 1.66–1.53 (t), 1.25 (s), 0.87–0.84 (t) ; UV/vis (DMSO): λ_max_ = 271, 369 nm.

*Bacterial Strains:* For the antibacterial experiments, the strains of *Staphylococcus epidermidis* (ATCC^®^ 14990™), *S. aureus* subsp. *aureus* (ATCC^®^ 25923™), and *P. aeruginosa PAO1* and *Escherichia coli* were used.

*Effects of **SLS@TC***, ***CTAB@TC***, ***TC***, ***SLS***, *and **CTAB** on the growth of microbial strains*: This study was performed according to standard procedure, which is also described elsewhere [21,22,23,24,25,26,27,28,29]. Briefly, the bacterial strains were streaked onto trypticase soy agar. The plates were incubated for 18–24 h at 37 °C. Three to five isolated colonies were selected of the same morphological appearance from the fresh agar plate using a sterile loop and transferred into a tube containing 2 mL of sterile saline solution. The optical density at 620 nm is 0.1, which corresponds to 10^8^ cfu/mL. The final inoculum size for broth dilution is 5 × 10^5^ cfu/mL. The total volume of the culture solution treated by micelles, **TC**, and surfactants, as well as the total volume of the positive and negative control, was 2 mL. The range of concentrations of micelles, TC, and surfactants is 0.02–300 μΜ. The growth is assessed after incubation for 20 h. The minimal inhibitory concentration (MIC) was determined as the concentration of the compound that inhibits the visible growth of the bacterium being investigated. The optical density of the solution at 620 nm versus concentrations graph leads to the MIC determination [21,22,23,24,25,26,27,28,29].

*Minimum bactericidal concentration testing:* This study was performed according to standard procedure, which is also described elsewhere [21,22,23,24,25,26,27,28,29]. Bacteria were initially cultivated in the presence of micelles, TC, and surfactants in broth culture for 20 h. The MBC values were determined in duplicate, by subculturing 4 μL of the broth an agar plate [21,22,23,24,25,26,27,28,29]. Colony growth suggests non-bactericidal activity of a metallodrug. Thus, the minimum bactericidal concentration was then defined as the lowest concentration at which a tested compound completely inhibits the microbes’ growth [21,22,23,24,25,26,27,28,29].

*Determination of the inhibition zone (IZ) through the agar disk-diffusion method*: This study was performed according to standard procedure, which is also described elsewhere [21,22,23,24,25,26,27,28,29]. Thus, agar plates were inoculated with a standardized inoculum (10^8^ cfu/mL) of the tested micro-organism. Filter paper disks (9 mm in diameter), which were previously soaked by micelles, **TC**, and surfactants (1 mM), were placed on the agar surface. The Petri dishes were incubated for 20 h and then the diameters of the inhibition zones were measured [21,22,23,24,25,26,27,28,29].

*Effects on biofilm formation by micelles:* Bacteria with a density of 6.7 × 10^6^ cfu mL^−1^ were inoculated into LB medium for *P. aeruginosa* or tryptic soy broth for *S. aureus* (total volume = 1500 μL) and cultured for 24 h at 37 °C. Afterwards, the content of each tube was carefully removed and the tubes were washed with 1 mL 0.9% saline dilution. The negative control contained broth only. After the bacteria were incubated with 50–600 μΜ of the micelles for 20 h at 37 °C, the content of each tube was aspirated; each tube was washed three times with 1 mL methanol and 2 mL 0.9% saline and left to dry. Then, the tubes were stained for 15 min with crystal violet solution (0.3% *w/v*). Excess stain was rinsed off with 1 mL methanol and 2 mL 0.9% saline solution and then 3 mL 0.9% saline solution. The tubes were left to dry for 24 h and the bounded crystal violet was released by adding 30% glacial acetic acid. The optical density of the solution yielded was then measured at 550 nm to give the biofilm biomass.

*Sulforhodamine B assay.* These studies were performed in accordance with the previously reported method [21,22,23,24,25,26,27,28,29]. Briefly, HCECs were seeded in a 96-well plate at a density of 10,000 cells and, after 24 h of cell incubation, the compounds were added in the concentration range of 5–40 μM for **SLS@TC**, 0.01–0.5 μΜ for **CTAB@TC**, 15–60 μΜ for **TC** and **SLS**, and 1–12 μΜ for **SLS**. HCECs were exposed with micelles, **TC**, and surfactants in IC_50_ values for a period of 48 h.

*Evaluation of genotoxicity by micronucleus assay.* The micronucleus assay was carried out as previously described [21,22,23,24,25,26,27,28,29]. The procedure is described here briefly. HCECs were seeded (at a density of 15 × 10^4^ cells/well cells per well) in glass cover slips, which were afterwards placed in six-well plates, with 3 mL of cell culture medium, and incubated for 24 h. HCECs were exposed with micelles at their IC_50_ values for a period of 48 h. Afterwards, the coverslips were washed three times with Phosphate Buffer Solution (PBS), and with a hypotonic solution (75 mM KCl) for 10 min at room temperature. The hypotonized cells were fixed by at least three changes of 1/3 acetic acid/ethanol. The coverslips were also washed with cold methanol containing 1% acetic acid. The coverslips were stained with acridine orange (5 μg mL^−1^) for 15 min at 37 °C. After, the coverslips were rinsed three times with PBS to remove any excess acridine orange stain. The number of micronucleated cells per 1000 cells was determined using a fluoresce microscope. The MN were identified according to the previously established criteria: (1) the area of MN should correspond to 1/256 and 1/9 of the area of the main nuclei; (2) the shape of MN should be round or oval; (3) the MN should be readily distinguished from artifacts; (4) MN are not linked or connected to the main nucleus; (5) MN may touch, but not overlap the main nuclei, and the micronuclear boundary should be distinguishable from the nuclear boundary; and (6) the MN have the same staining intensity as the main nuclei [24].

*Evaluation of toxicity with brine shrimp assay*: Brine shrimp assay was performed by a method previously described [28]. Here, 1 g cysts were initially hydrated in freshwater for one hour in a separating funnel or cone shaped container. Seawater was prepared by dissolving 17 g of sea salt in 500 mL of distilled water [28]. The cone was facilitated with good aeration for 48 h at room temperature and under continuous illumination. After hatching, nauplii released from the egg shells were collected at the bright side of the cone (near the light source) using a micropipette. The larvae were isolated from the eggs by aliquoting them in a small beaker containing NaCl 0.9% [28]. An aliquot (0.1 mL) containing about 5 to 15 nauplii was introduced to each well of a 24-well plate and micelles were added in each well at MIC^min^, MIC^max^, 2 × MIC^max^, and 3 × MIC^max^. The final volume of each well is 1 mL with NaCl 0.9%. The brine shrimps were observed after of 24 h using a stereoscope. Larvae are considered dead if they do not exhibit any internal or external movement in 10 s of observation. Each experiment was repeated three times.

## Figures and Tables

**Table 1:** Correlation diagram for moderate and severe distortions of tetrahedral species with T_d_ symmetry to distorted tetrahedral species with C_3ν_, C_2ν_, and C_s_ symmetry, respectively.

T_d_	C_3ν_	C_2ν_	C_s_	C_1_
ν_1_(Α_1_) 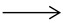	ν_1_(Α_1_) 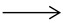	ν_1_(Α_1_) 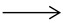	ν_1_(Α’) 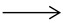	ν_1_(Α)
ν_2_(Ε) 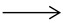	ν_2_(Ε) 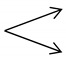	ν_2a_(Α_1_) 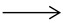 ν_2b_(Α_2_) 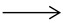	ν_2a_(Α’) 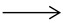 ν_2b_(Α”) 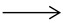	ν_2a_(Α) ν_2b_(Α)
ν_3_(F_2_) 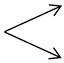	ν_3a_(Α_1_) 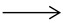 ν_3b_(E) 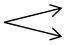	ν_3a_(Α_1_) 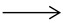 ν_3b_(B_1_) 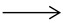 ν_3c_(B_2_) 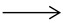	ν_3__a_(Α’) 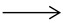 ν_3__b_(Α’) 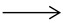 ν_3__c_(A”) 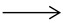	ν_3a_(Α) ν_3b_(Α) ν_3c_(A)
ν_4_(F_2_) 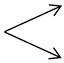	ν_4a_(Α_1_) 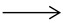 ν_3b_(E) 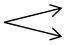	ν_4a_(Α_1_) 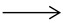 ν_4b_(B_1_) 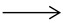 ν_4_(B_2_) 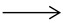	ν_4__a_(Α’) 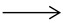 ν_4__b_(Α’) 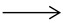 ν_4__c_(A”) 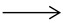	ν_4a_(Α) ν_4b_(Α) ν_4c_(A)

**Table 2:** Minimum inhibitory concentrations (MICs), minimum bactericidal concentrations (MBCs), inhibition zones (IZs), and biofilm elimination concentration (BEC) of **SLS@TC**, **CTAB@TC**, **TC**, **SLS**, and **CTAB** against *P. aeruginosa*, *E. coli*, *S. epidermidis* and *S. aureus*. Their IC_50_ values against human corneal epithelial cells (HCECs). TC, tetracycline; SLS, sodium lauryl sulfate; CTAB, cetrimonium bromide.

Sample	*P. aeruginosa*	*E. coli*	*S. epidermidis*	*S. aureus*	Ref.
	MIC (μΜ)				
**SLS@TC**	27.20 ± 1.60	4.07 ± 0.30	6.22 ± 0.80	3.70 ± 0.82	*
**CTAB@TC**	5.80 ± 0.41	0.13 ± 0.002	0.07 ± 0.01	0.15 ± 0.04	*
**TC**	28.60 ± 4.90	5.60 ± 1.33	37.80 ± 14.06	1.32 ± 0.34	*
**SLS**	>250	39.80 ± 0.60	49.70 ± 0.40	42.90 ± 6.20	[27]
**CTAB**	>300	5.08 ± 1.11	0.36 ± 0.09	1.95 ± 0.36	
	MBC (μΜ)				
**SLS@TC**	87.10 ± 6.90	116.6 ± 20.60	12.00 ± 4.00	9.10 ± 1.10	*
**CTAB@TC**	95.0 ± 5.70	12.0 ± 4.50	1.30 ± 0.40	0.50 ± 0.10	*
**TC**	92.0 ± 18.00	150.0 ± 25.30	171.40 ± 29.10	10.0 ± 0.0	*
**SLS**	>250	>250	100	60	[27]
**CTAB**	>300	150.0 ± 40.0	5.30 ± 0.70	10.0 ± 0.0	
	MBC/MIC				
**SLS@TC**	3.19	28.64	1.92	2.45	*
**CTAB@TC**	16.26	92.30	18.57	3.33	*
**TC**	3.21	26.60	4.53	7.57	*
**SLS**	ND	ND	2.01	1.39	[27]
**CTAB**		29.52	14.70	5.12	
	IZ (mm)				
**SLS@TC**	23.8 ± 1.2	29.3 ± 3.4	31.8 ± 3.1	30.5 ± 1.0	*
**CTAB@TC**	13.7 ± 0.3	22.7 ± 0.9	20.0 ± 1.5	34.0 ± 2.0	*
**TC**	15.2 ± 1.4	22.5 ± 0.6	11.8 ± 0.5	34.3 ± 1.8	*
**SLS**	ND	ND	ND	ND	[27]
**CTAB**	ND	12.0 ± 0.6	14.3 ± 1.3	14.3 ± 0.7	*
	BEC (μΜ)				
**SLS@TC**	557 ± 111	-	-	1913 ± 380	*
**CTAB@TC**	1875 ± 614	-	-	983 ± 204	*
**TC**	427 ± 84	-	-	>2304	[53]
	IC_50_ (μΜ) HCEC cells				
**SLS@TC**	14.1 ± 0.40				*
**CTAB@TC**	0.09 ± 0.01				*
**TC**	>60				*
**SLS**	>60				*
**CTAB**	3.7 ± 0.10				*

* In this work, ND = no inhibitory zone (IZ) was developed.

**Table 3:** Survival of *Artemia salina* larvae in MIC^min^, MIC^max^, 2 × MIC^max^, and 3 × MIC^max^ of **SLS@TC** and **CTAB@TC** solutions after 24 h.

Micelle	MIC^min^	MIC^max^	2 × MIC^max^	3 × MIC^max^
**SLS@TC**	78.6 ± 15.2	79.5 ± 15.0	50.0 ± 14.0	59.1 ± 8.9
**CTAB@TC**	93.8 ± 12.2	62.5 ± 24.5	75.0 ± 49.0	71.7 ± 22.9

## Supplementary Materials

The following are available online at https://www.mdpi.com/2079-6382/9/12/845/s1, Figure S1. Conductance vs. [**SLS**]/[**TC**] (A) and [**CTAB**]/[**TC**] (B) diagrams for CMC determination; Figure S2. TD/DTA graph of **SLS@TC** (A) and **CTAB@TC** (B); Figure S3. Diagram of DSC **SLS@TC** and **CTAB@TC**; Figure S4. IR spectra of **TC**, **SLS**, and **SLS@TC**; Figure S5. IR spectra of **TC**, **CTAB**, and **CTAB@TC**; Figure S6 (A) UV spectra of TC in DMSO at 1 × 10^−4^, 8 × 10^−5^, 6 × 10^−5^, 4 × 10^−5^, 2 × 10^−5^, and 1 × 10^−5^ M respectively; (B) absorbance of **TC** solution in DMSO at λ_max_ = 269 nm vs. concentration linear graph; Figure S7. Minimum inhibitory concentration of **SLS@TC** against *P. aeruginosa* (A), *E. coli* (B), *S. epidermidis* (C), and *S. aureus* (D); Figure S8. Minimum inhibitory concentration of **CTAB@TC** against *P. aeruginosa* (A), *E. coli* (B), *S. epidermidis* (C), and *S. aureus* (D); Figure S9. Minimum inhibitory concentration of TC against *P. aeruginosa* (A), *E. coli* (B), *S. epidermidis* (C), and *S. aureus* (D); Figure S10. Minimum inhibitory concentration of **CTAB** towards *P. aeruginosa* (A), *E. coli* (B), *S. epidermidis* (C), and *S. aureus* (D); Figure S11. Minimum bactericidal concentration of **SLS@TC** towards *P. aeruginosa* (A), *E. coli* (B), *S. epidermidis* (C), and *S. aureus* (D); Figure S12. Minimum bactericidal concentration of **CTAB@TC** towards *P. aeruginosa* (A), *E. coli* (B), *S. epidermidis* (C), and *S. aureus* (D); Figure S13. Minimum bactericidal concentration of TC towards *P. aeruginosa* (A), *E. coli* (B), *S. epidermidis* (C), and *S. aureus* (D); Figure S14. Minimum bactericidal concentration of **CTAB** towards *P. aeruginosa* (A), *E. coli* (B), *S. epidermidis* (C), and *S. aureus* (D); Figure S15. Biofilms of **SLS@TC** towards *P. aeruginosa* (A) and *S. aureus* (B); Figure S16. Biofilms of **CTAB@TC** towards *P. aeruginosa* (A) and *S. aureus* (B); Figure S17. Biofilms of **TC** towards *P. aeruginosa.*
