# Supplementary material for: Tetracycline Water Soluble Formulations with Enhanced Antimicrobial Activity
Source: Antibiotics (Basel). 2020 Nov 26;9(12):845. doi: 10.3390/antibiotics9120845 (PMC7760183; doi:10.3390/antibiotics9120845)
Supplement: Supplementary file 1 [file antibiotics-09-00845-s001.pdf]

antibiotics

MDPI

Article

Tetracycline Water Soluble Formulations with Enhanced Antimicrobial Activity

A. Meretoudi [1], C. N. Banti [1,*], P. Siafarika [2], A. G. Kalampounias [2,3,*] and S. K. Hadjikakou [1,3,*]

[1] Inorganic Chemistry laboratory, Department of Chemistry, University of Ioannina, 45110 Ioannina, Greece; ameretoudi1996@gmail.com

[2] Physical Chemistry Laboratory, Department of Chemistry, University of Ioannina, 45110 Ioannina, Greece; pch1246@uoi.gr

[3] University Research Center of Ioannina (URCI), Institute of Materials Science and Computing, 45110 Ioannina, Greece

* Correspondence: cbanti@uoi.gr (C.N.B.); akalamp@uoi.gr (A.G.K.); shadjika@uoi.gr (S.K.H.); Tel.: +30-26510-08374 (S.K.H.)

Received: 19 October 2020; Accepted: 23 November 2020; Published: 26 November 2020

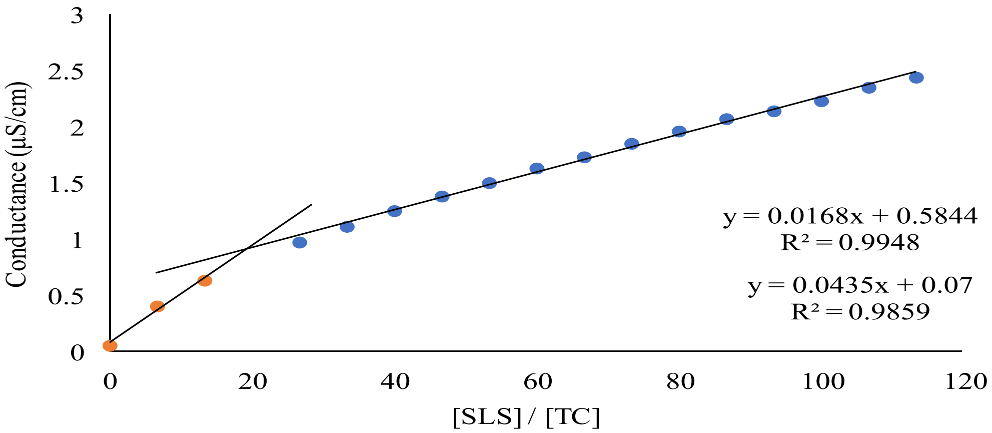

(**A**)

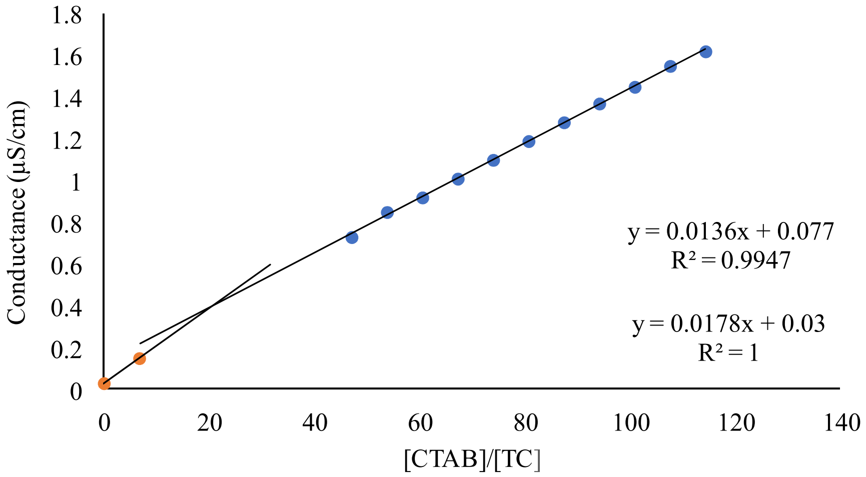

(**B**)

Figure S1. Conductance vs [SLS]/[TC] (**A**) and [CTAB]/[TC] (**B**) diagrams for CMC determination.

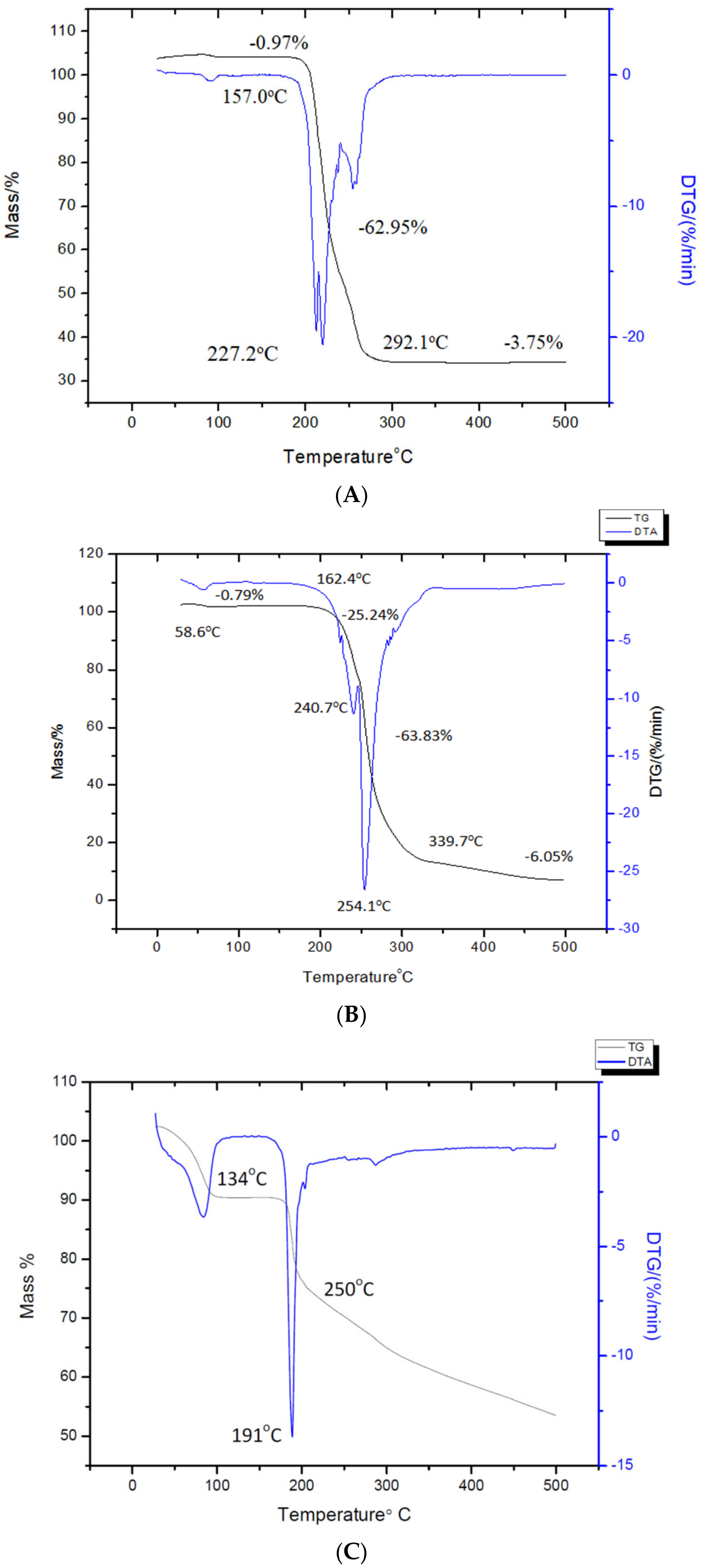

Figure S2. TD/DTA graph of **SLS@TC** (**A**), **CTAB@TC** (**B**) and **TC** (**C**).

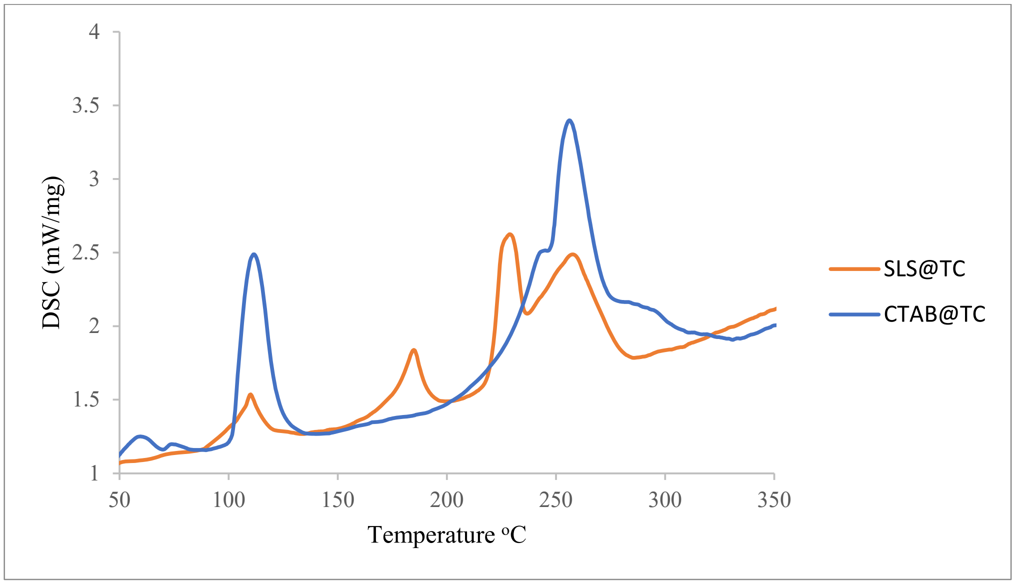

Figure S3. Diagram of DSC SLS@TC and CTAB@TC.

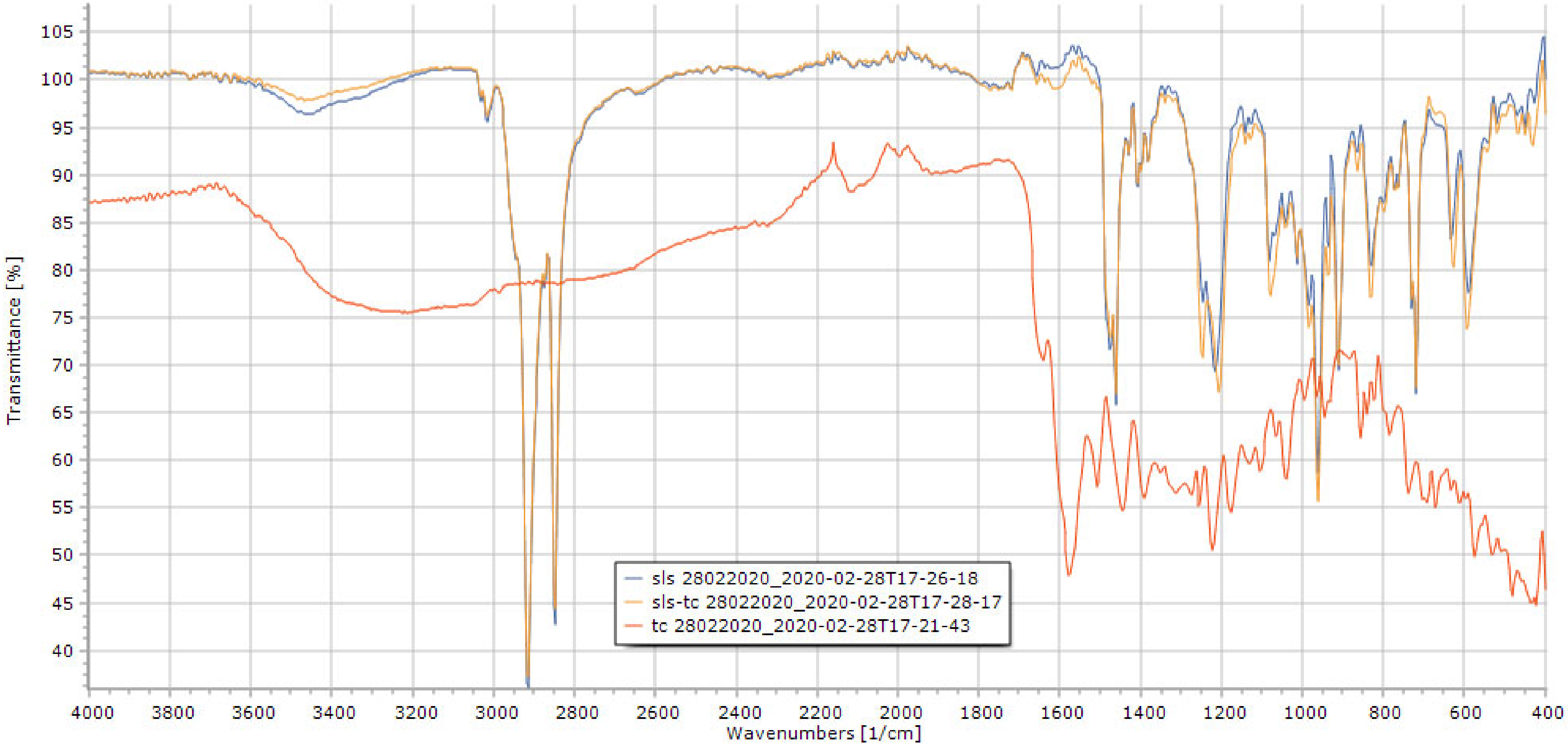

Figure S4. IR spectra of TC, SLS, **SLS@TC.**

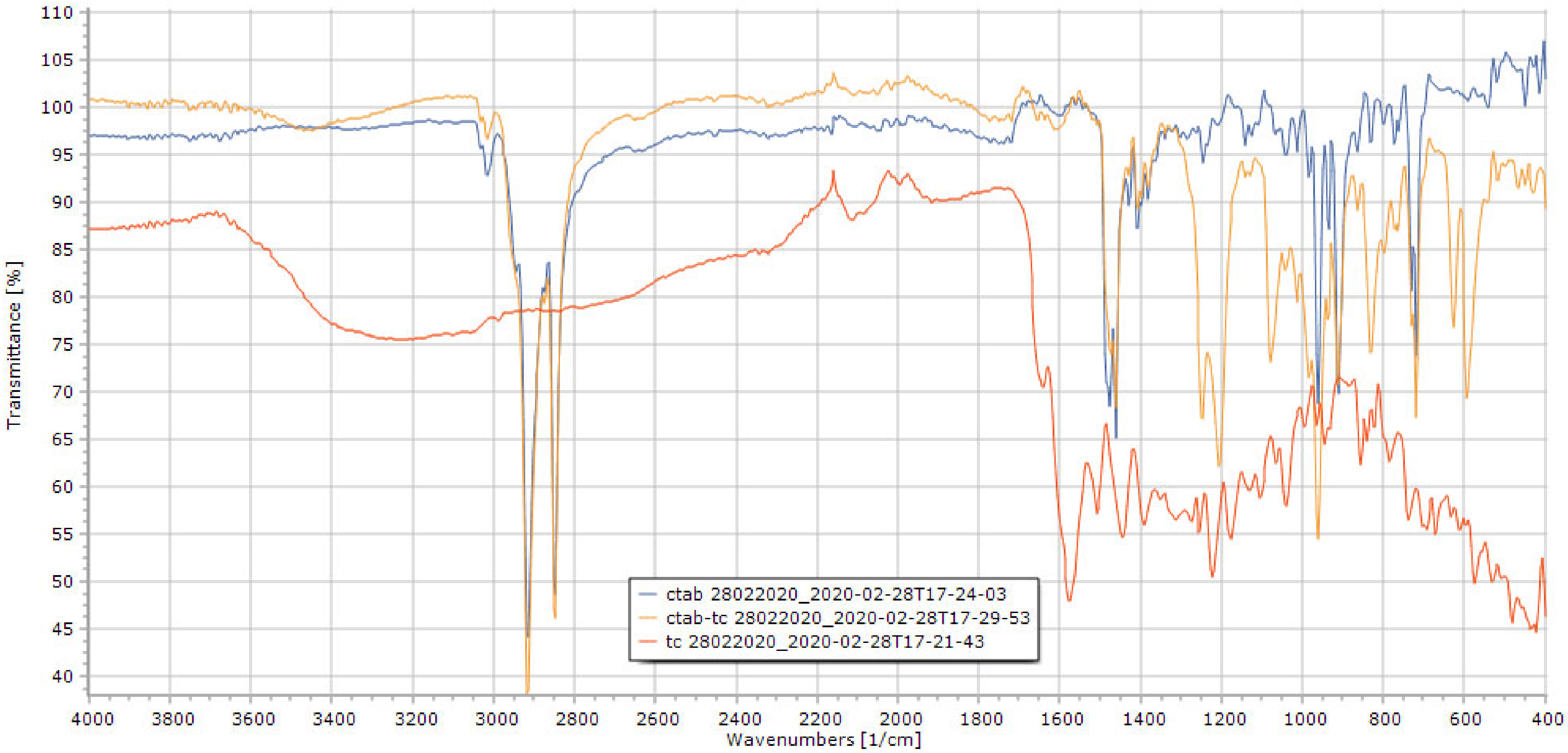

Figure S5. IR spectra of TC, CTAB, **CTAB@TC.**

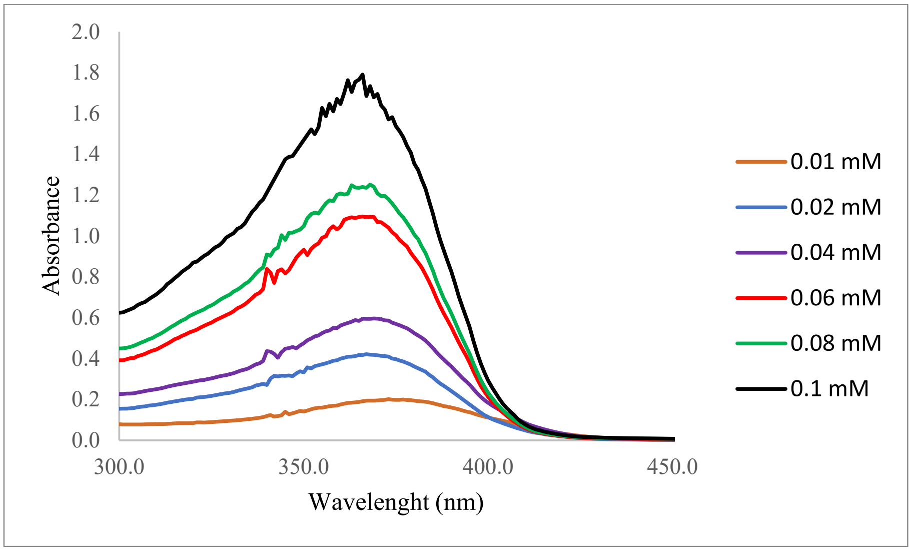

(**A**)

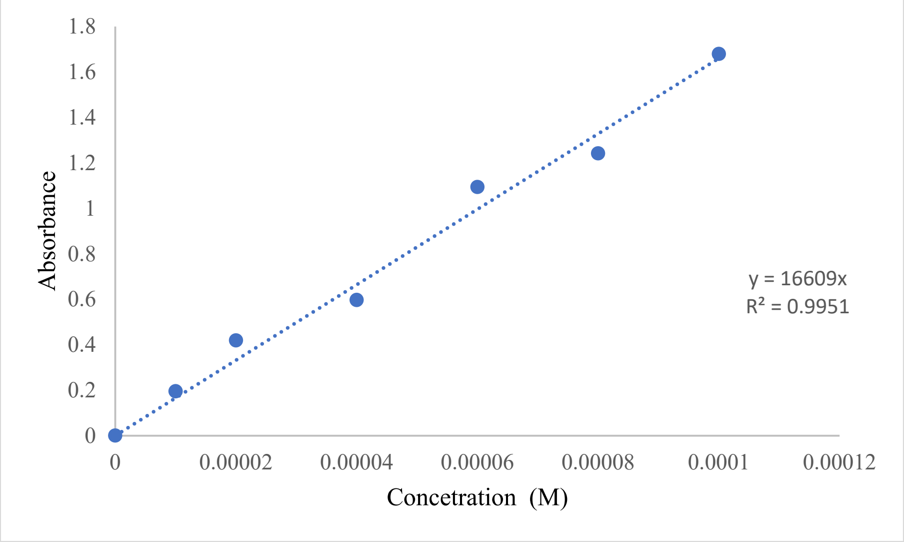

(**B**)

Figure S6. (**A**) UV spectra of TC in DMSO at 1 × 10^{-4}, 8 × 10^{-5}, 6 × 10^{-5}, 4 × 10^{-5}, 2 × 10^{-5}, and 1 × 10^{-5} M respectively (**B)** Absorbance of TC solution in DMSO at λ_{max}= 269 nm *vs* Concentration linear graph.

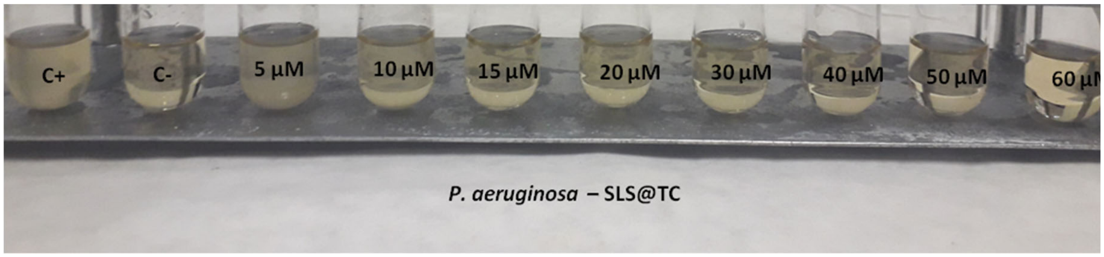

(**A**)

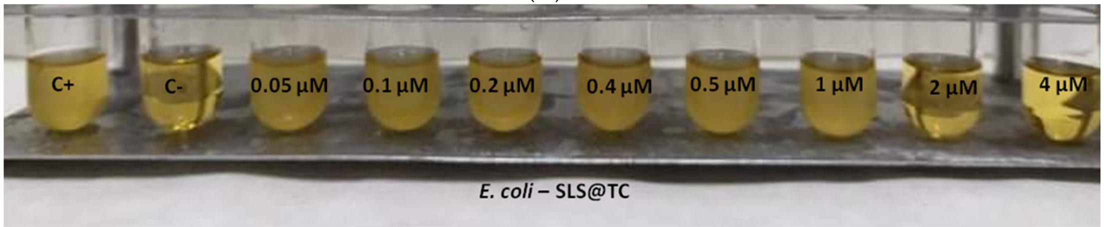

(**B**)

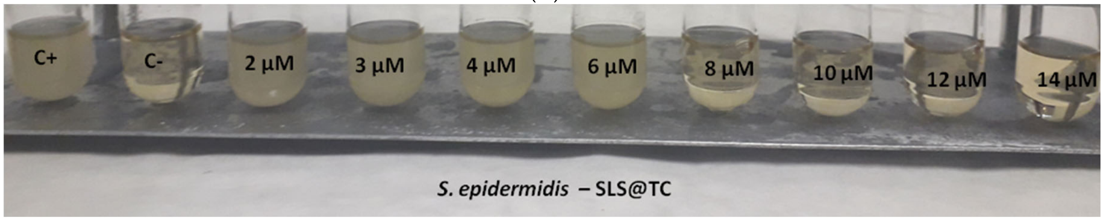

(**C**)

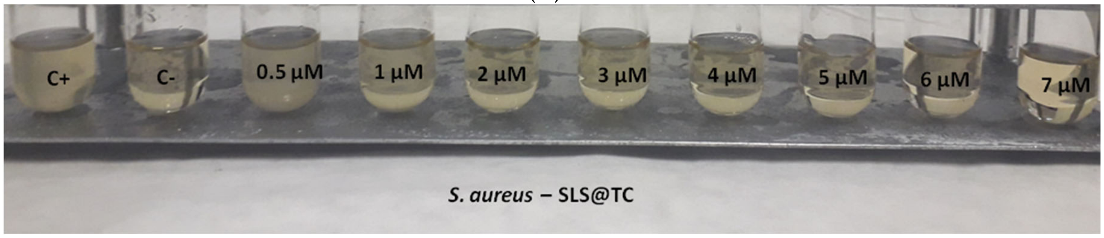

(**D**)

Figure S7. Minimum Inhibitory Concentration of **SLS@TC** against *P. aeruginosa* (**A**), against *E. coli* (**B**), against *S. epidermidis* (**C**), and *S. aureus* (**D**).

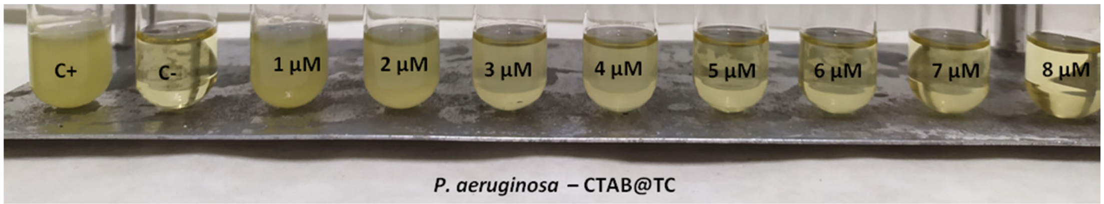

(**A**)

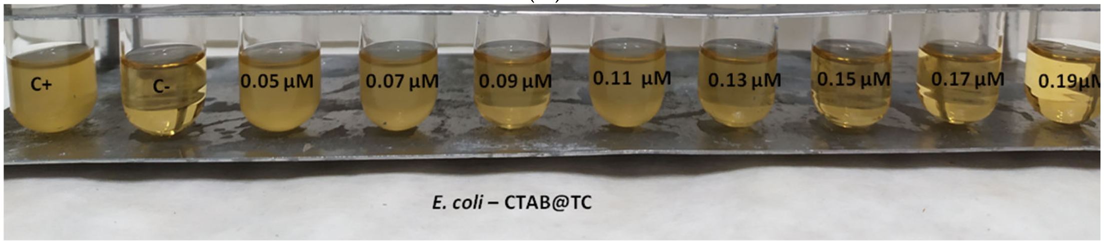

(**B**)

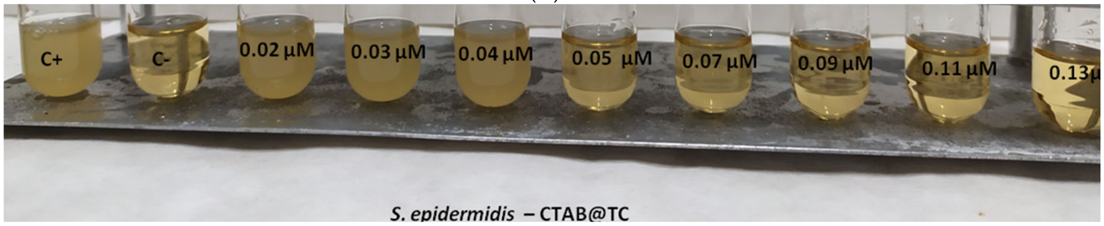

(**C**)

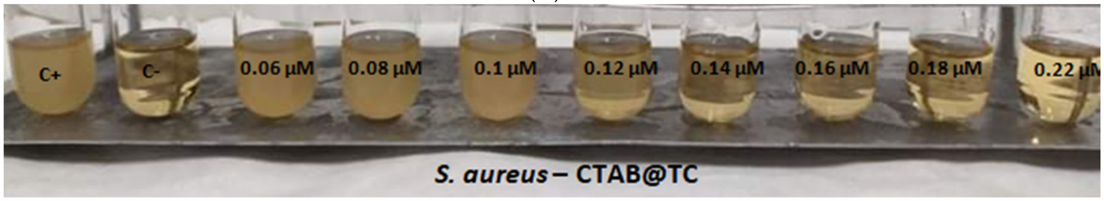

(**D**)

Figure S8. Minimum Inhibitory Concentration of **CTAB@TC** against *P. aeruginosa* (**A**), against *E. coli* (**B**), against *S. epidermidis* (**C**), and *S. aureus* (**D**).

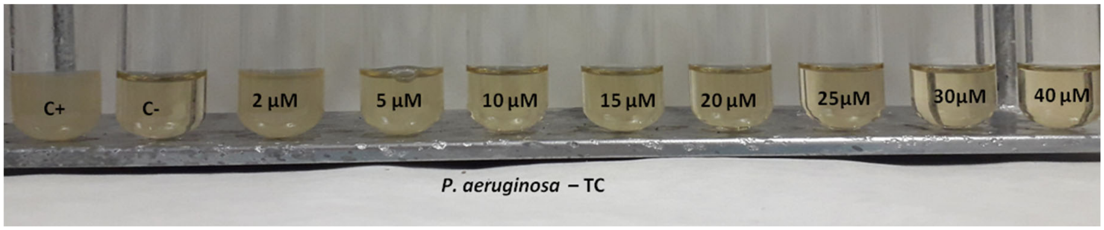

(**A**)

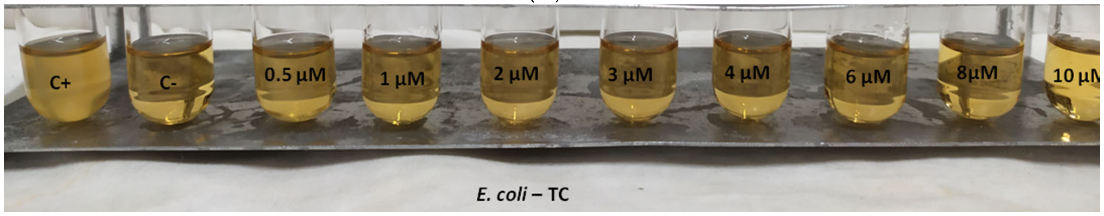

(**B**)

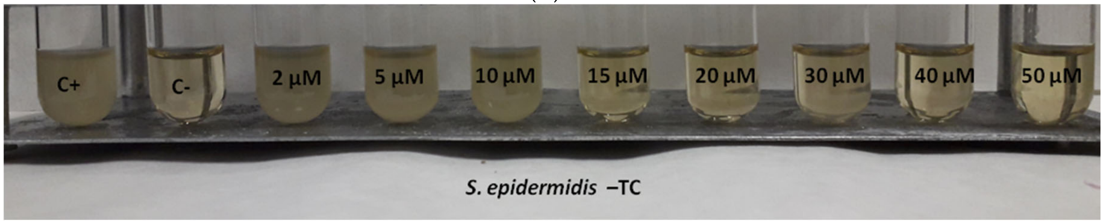

(**C**)

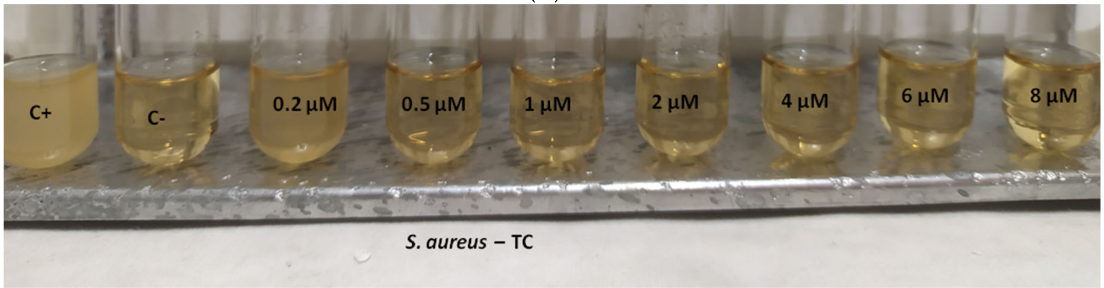

(**D**)

Figure S9. Minimum Inhibitory Concentration of TC against *P. aeruginosa* (**A**), against *E. coli* (**B**), against *S. epidermidis* (**C**), and *S. aureus* (**D**).

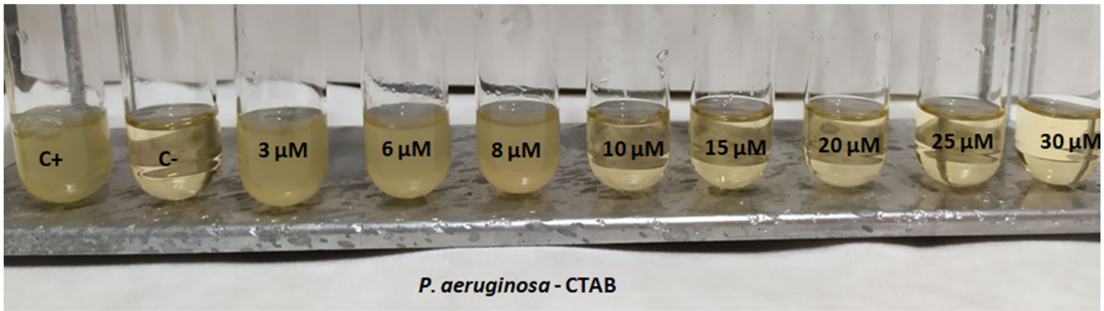

(**A**)

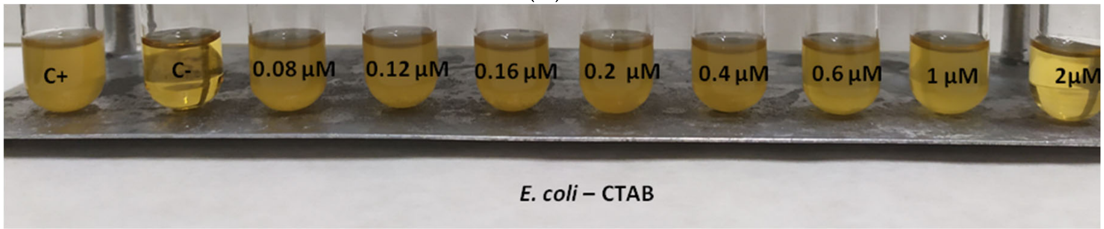

(**B**)

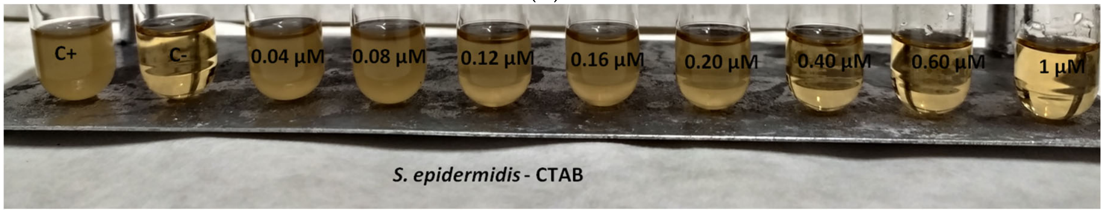

(**C**)

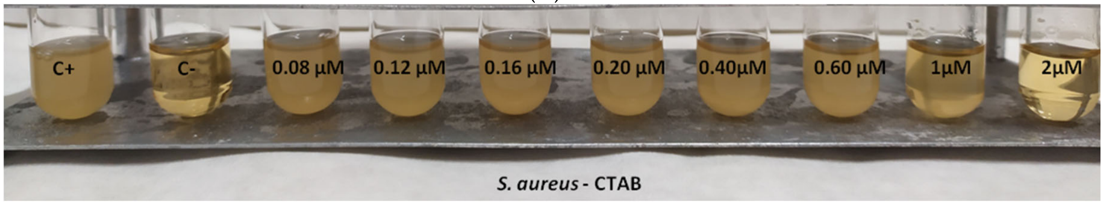

(**D**)

Figure S10. Minimum Inhibitory Concentration of CTAB towards *P. aeruginosa* (**A**), against *E. coli* (**B**), against *S. epidermidis* (**C**), and *S. aureus* (**D**).

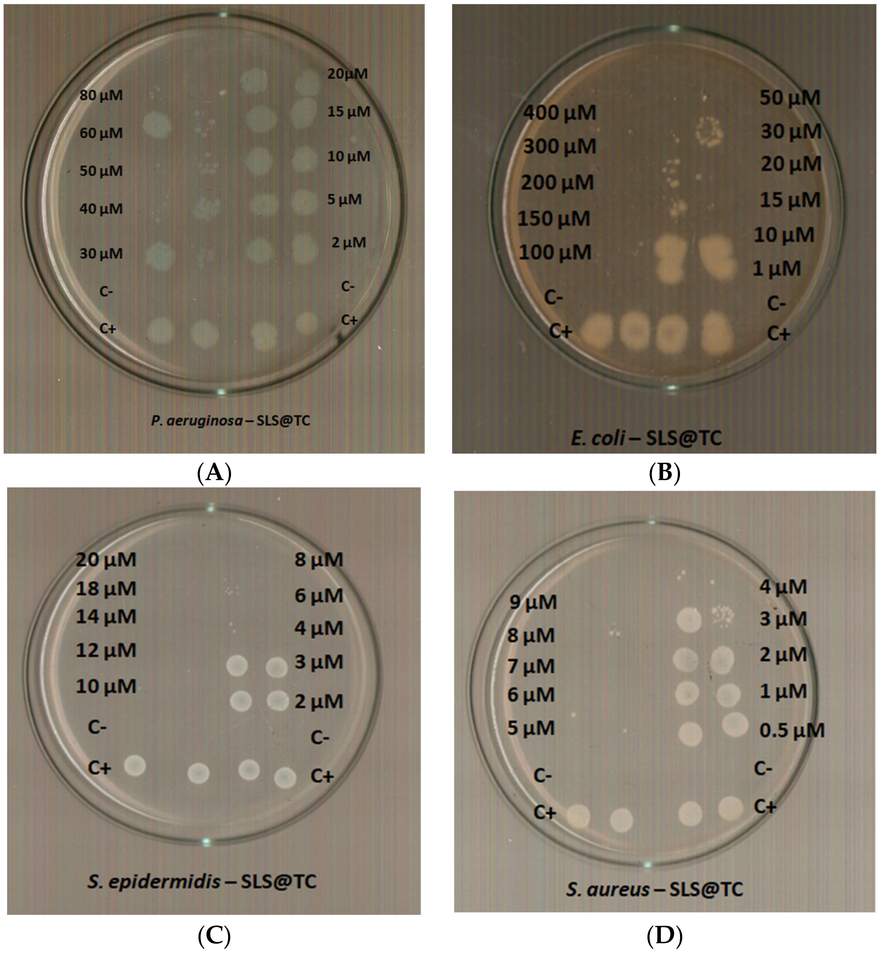

Figure S11. Minimum bactericidal concentration of **SLS@TC** towards *P. aeruginosa* (**A**), *E. coli* (**B**), *S. epidermidis* (**C**) and *S. aureus*. (**D**).

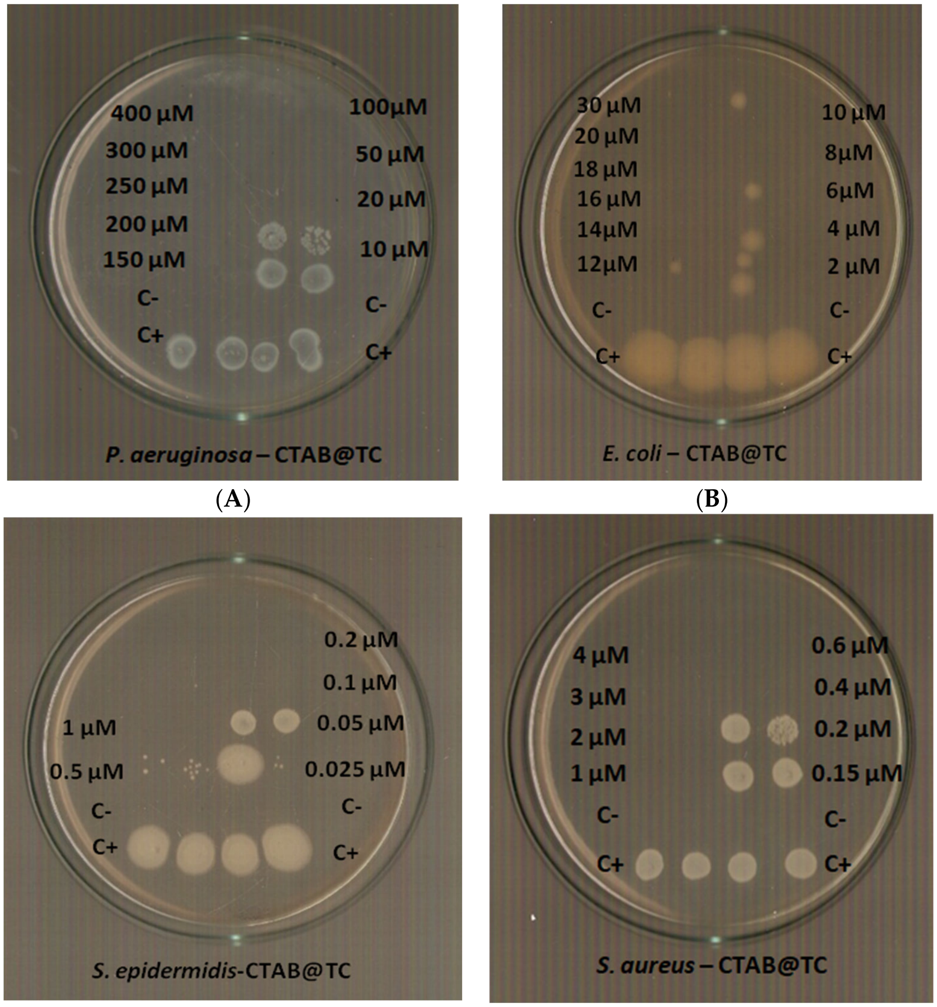

(**A**) (**B**)

(**C**) (**D**)

Figure S12. Minimum bactericidal concentration of **CTAB@TC** towards *P. aeruginosa* (**A**), *E. coli* (**B**), *S. epidermidis* (**C**) and *S. aureus*. (**D**).

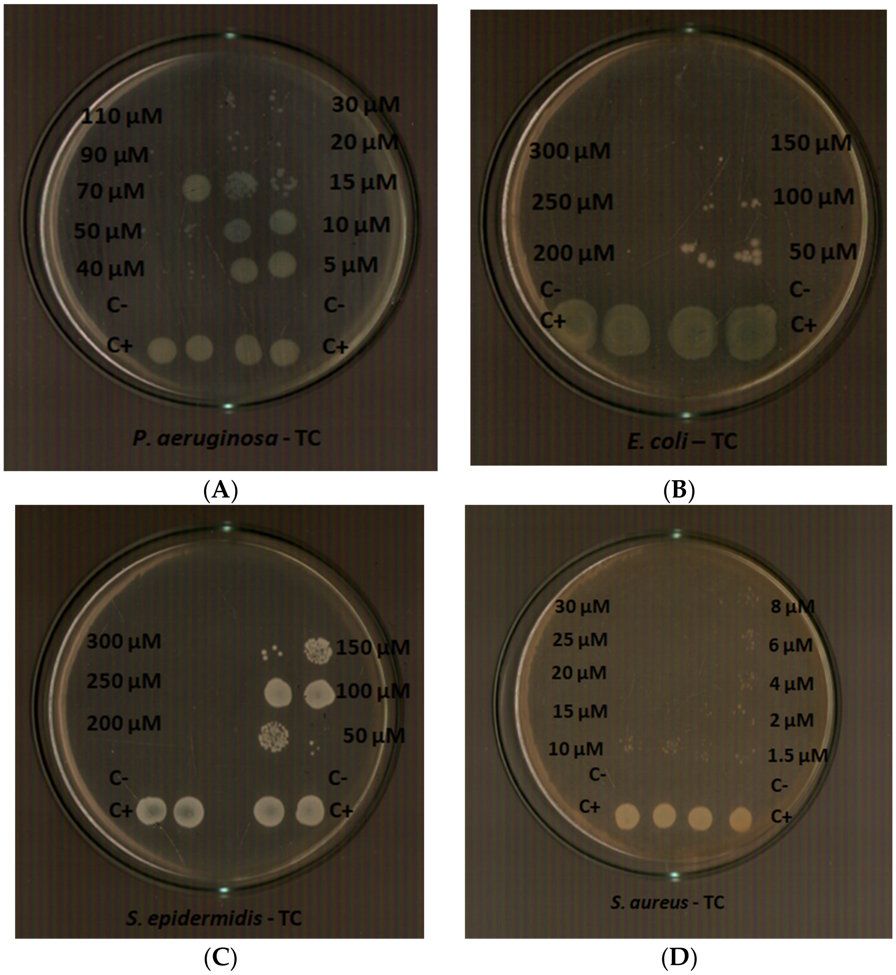

Figure S13. Minimum bactericidal concentration of **TC** towards *P. aeruginosa* (**A**), *E. coli* (**B**), *S. epidermidis* (**C**) and *S. aureus*. (**D**).

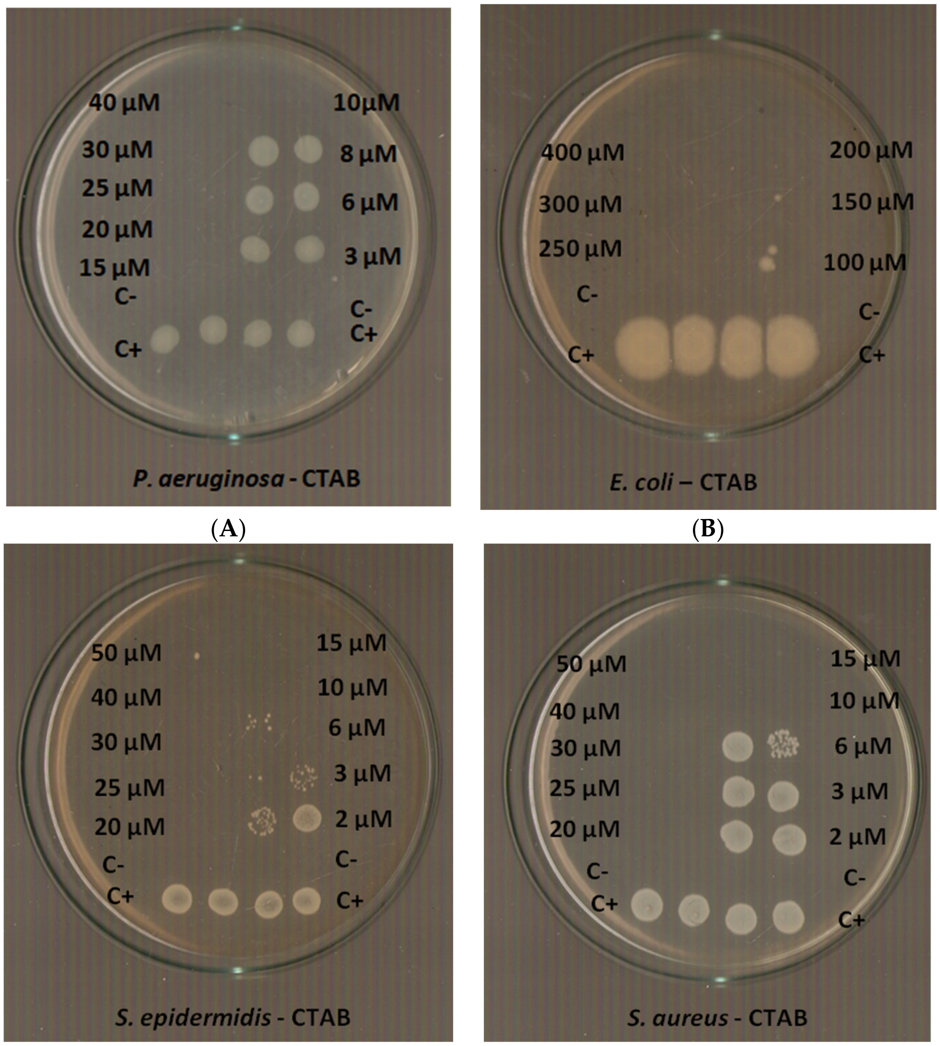

(**A**) (**B**)

(**C**) (**D**)

Figure F14. Minimum bactericidal concentration of **CTAB** towards *P. aeruginosa* (A), *E. coli* (B), *S. epidermidis* (C) and *S. aureus*. (D).

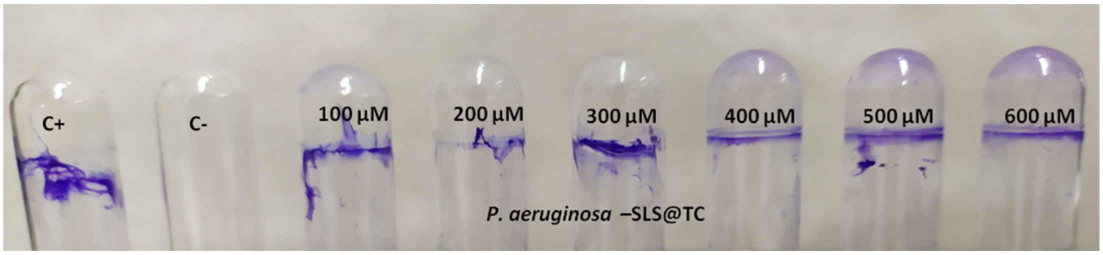

(**A**)

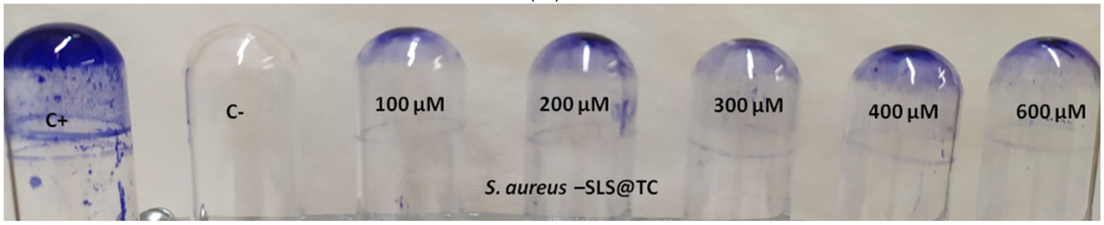

(**B**)

Figure S15. Biofilms of **SLS@TC** towards *P. aeruginosa* (**A**), and *S. aureus*. (**B**).

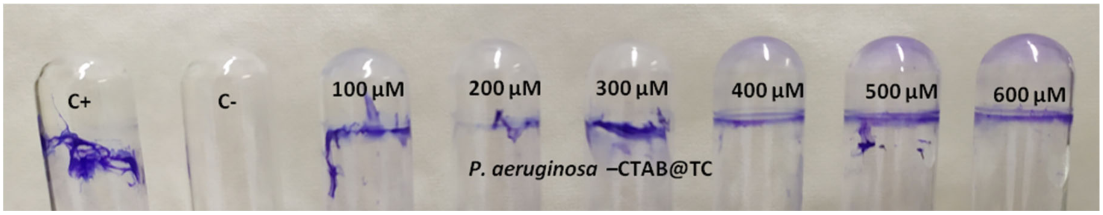

(**A**)

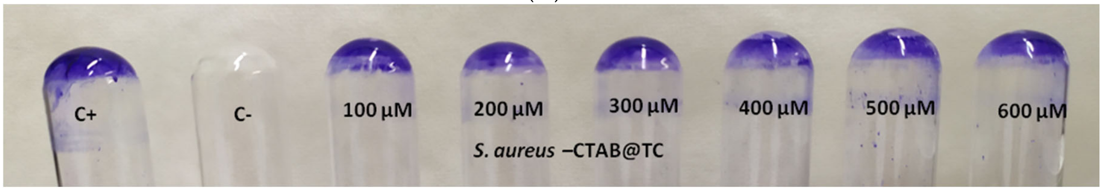

(**B**)

Figure S16. Biofilms of **CTAB@TC** towards *P. aeruginosa* (**A**), and *S. aureus*. (**B**).

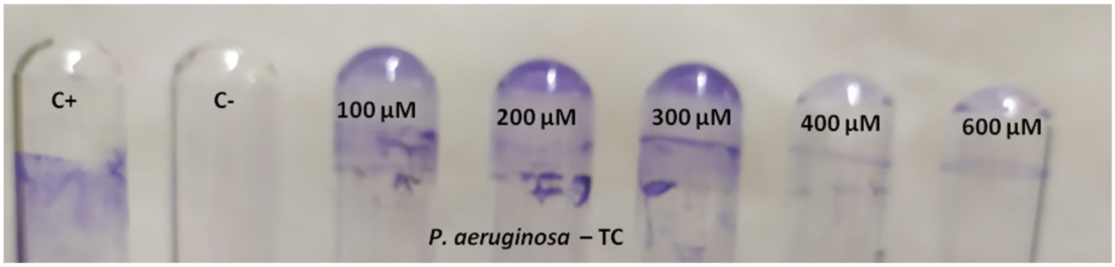

Figure S17. Biofilms of **TC** towards *P. aeruginosa.*
